# Resolution of MALDI-TOF compared to whole genome sequencing for identification of *Bacillus* species isolated from cleanrooms at NASA Johnson Space Center

**DOI:** 10.3389/fmicb.2025.1499516

**Published:** 2025-04-09

**Authors:** Farnaz Mazhari, Aaron B. Regberg, Christian L. Castro, Michael G. LaMontagne

**Affiliations:** ^1^Department of Biology and Biotechnology, University of Houston-Clear Lake, Houston, TX, United States; ^2^Jacobs, JETS II Contract, NASA Johnson Space Center, Houston, TX, United States; ^3^Astromaterials Research and Exploration Science (ARES) Division, NASA Johnson Space Center, Houston, TX, United States; ^4^JES Tech, NASA Johnson Space Center, Houston, TX, United States

**Keywords:** built, MALDI-TOF, whole genome sequencing, cleanroom, *Bacillus*, NASA, astromaterials

## Abstract

**Introduction:**

Bacteria are frequently isolated from surfaces in cleanrooms, where astromaterials are curated, at NASA’s Lyndon B. Johnson Space Center (JSC). *Bacillus* species are of particular interest because endospores can endure extreme conditions. Current monitoring programs at JSC rely on culturing microbes from swabs of surfaces followed by identification by 16S rRNA sequencing and the VITEK 2 Compact bacterial identification system. These methods have limited power to resolve *Bacillus* species. Whole genome sequencing (WGS) is the current standard for bacterial identification but is expensive and time-consuming. Matrix-assisted laser desorption - time of flight mass spectrometry (MALDI-TOF MS), provides a rapid, low-cost, method of identifying bacterial isolates and has a higher resolution than 16S rRNA sequencing, particularly for *Bacillus* species; however, few studies have compared this method to WGS for identification of *Bacillus* species isolated from cleanrooms.

**Methods:**

To address this, we selected 15 isolates for analysis with WGS and MALDI-TOF MS. Hybrid next-generation (Illumina) and 3rd-generation (nanopore) sequencing were used to draft genomes. Mass spectra, generated with MALDI-TOF MS, were processed with custom scripts to identify clusters of closely related isolates.

**Results:**

MALDI-TOF MS and WGS identified 13/15 and 9/14 at the species level, respectively, and clusters of species generated from MALDI-TOF MS showed good agreement, in terms of congruence of partitioning, with phylotypes generated with WGS. Pairs of strains that were > 94% similar to each other, in terms of average amino acid identity (AAI) predicted by WGS, consistently showed cosine similarities of mass spectra >0.8. The only discordance was for a pair of isolates that were classified as *Paenibacillus* species. This pair showed relatively high similarity (0.85) in terms of MALDI-TOF MS but only 85% similarity in terms of AAI. In addition, some strains isolated from cleanrooms at the JSC appeared closely related to strains isolated from spacecraft assembly cleanrooms.

**Discussion:**

Since MALDI-TOF MS costs less than whole genome sequencing and offers a throughput of hundreds of isolates per hour, this approach appears to offer a cost-efficient option for identifying *Bacillus* species, and related microbes, isolated during routine monitoring of cleanrooms and similar built environments.

## 1 Introduction

NASA has maintained cleanrooms at the Johnson Space Center (JSC) for curating extraterrestrial samples from the moon, meteorites, cosmic dust, asteroids, comets, solar wind particles, and micrometeorite impacts on space-exposed hardware starting with the lunar samples from the Apollo missions in 1969 (McCubbin et al., 2019). Oligotrophic, low humidity conditions, regular cleaning and air filtration render these facilities inhospitable to microbial life. Despite these fastidious controls, the cleanrooms contain bacteria and fungi (Regberg et al., 2018), which could alter the composition of astromaterials and confound searches for extraterrestrial life (Rummel, 2001). The presence of bacteria and fungi in the curation cleanrooms is acceptable because current astromaterials collections do not have biological contamination control requirements; however, future sample collections, such as the Mars sample return missions, will have these requirements (Carrier et al., 2021). To prepare for these missions, NASA has developed a routine microbial monitoring program for existing collections (McCubbin et al., 2019). *Bacillus* species comprise, on average, 45% of the microbes cultured in this curation program at the JSC (Mazhari, 2021) and are frequently isolated from cleanrooms at the NASA Jet Propulsion Laboratory (JPL) (Tirumalai et al., 2013; Tirumalai et al., 2018).

Strain-level identification of microbes recovered from cleanrooms is important for developing a robust microbial source tracking program and overall contamination control strategy (Song et al., 2024); however, 16S rRNA sequencing, which is widely used to identify bacterial isolates (Church et al., 2020), lacks the resolution required to differentiate closely related *Bacillus* species. For example, 16S rRNA gene sequences of many *Bacillus* species, that occupy fundamentally different environmental niches, are over 99% identical (Yamada et al., 1999). This limits the utility of 16S rRNA gene sequencing for monitoring diversity within curation cleanrooms (Espariz et al., 2016). Whole genome sequencing (WGS) has emerged as the definitive method for microbial identification (Kwong et al., 2015) and is widely used for tracking food-borne pathogens and disease outbreaks (Brown et al., 2019). However, building a library for microbial identification by WGS costs at least $400 per isolate (Brown et al., 2021) and requires highly trained personnel to generate and interpret the data.

Matrix-assisted laser desorption – time of flight mass spectrometry (MALDI-TOF MS) systems provide strain-level identification of microbes, for less than a dollar an isolate, in seconds (Gagné-Bourque et al., 2015). For example, MALDI-TOF can differentiate strains with different functional properties that produce and are resistant to antibiotics (Flores-Treviño et al., 2019) and differentiate between pathogenic and virulent strains of *Bacillus* species (Celandroni et al., 2016). MALDI-TOF MS systems use pattern matching between mass spectra generated from isolates and mass spectra generated from reference strains (Singhal et al., 2015). This high-throughput method is cheaper and more accurate than conventional biochemical systems for bacterial identification (Lévesque et al., 2015; Seng et al., 2009) and for identifying *Bacillus* species (Lasch et al., 2009) and related genera (Celandroni et al., 2016). MALDI-TOF MS appears comparable to WGS for identification of the specific pathogenic bacteria (da Silva et al., 2020; Nithimongkolchai et al., 2023; Rudolph et al., 2019; Werinder et al., 2021); however, these systems have limitations (Haider et al., 2023; Rychert, 2019). For example, MALDI-TOF MS systems can struggle to differentiate some species related to *Bacillus cereus* (Muigg et al., 2022) and lack of suitable reference spectra has limited the application of MALDI-TOF MS outside of the field of clinical microbiology (Rahi et al., 2016). Further, variation between strains of bacteria, can influence the performance of MALDI-TOF systems, so there is a need to evaluate this approach for bacteria isolated from different environments (Emami et al., 2016; Topić Popović et al., 2023). This need is particularly pressing for extreme environments (Kopcakova et al., 2014), including cleanrooms (da Costa et al., 2022) and similar facilities (Seuylemezian et al., 2018).

In this study, we compared the resolution of MALDI-TOF MS to WGS for a set of bacteria isolated from cleanrooms at the Johnson Space Center. Draft genomes of 14 bacteria were assembled with a hybrid of Illumina and Oxford Nanopore sequencing and genomic relationships were characterized using an estimated maximum-likelihood phylogenomic tree. The resolution of WGS, in terms of average amino acid identity, was then compared to MALDI-TOF MS with custom scripts following LaMontagne (LaMontagne et al., 2021). MALDI-TOF MS showed species-level resolution, which is comparable to WGS.

## 2 Materials and methods

### 2.1 Sample collection and genus-level identification

As part of routine microbial monitoring of cleanrooms, samples were collected from the Meteorite, Cosmic Dust, Star Dust, Lunar, Genesis, and Hayabusa labs, and a temporary Cold Curation facility at JSC (Mazhari, 2021). Puritan Brand, Sterile, DNA Free, Foam-Tipped-Applicator (Part Number: 25–1805 1PF RND FDNA) and Puritan Brand, Sterile, Polyester-Tipped-Applicators (Part Number: 25–1000 1PD) were used to sample 300 cm^2^ areas of cleanroom surfaces described in Table 1. Air sampling of the meteorite lab was conducted using the SAS Super 180 air sampler at 180 L/ min for 2 min. (360 L). using one Tryptic Soy agar (TSA) and one Sabouraud Dextrose agar (SDA) plate. Samples were transported to a dedicated microbiology lab and the swabs were resuspended in 15 mL of phosphate-buffered saline (PBS) and vortexed for 10 s. Four Tryptic soy agar (TSA), two Blood agar (BA) were inoculated with 100 μL PBS suspensions. Two Reasoner’s 2A agar (R2A), two Sabouraud Dextrose agar (SDA), one Sabouraud Dextrose Chloramphenicol agar (SDA + C), and one Potato Dextrose agar (PDA) plates were inoculated with a 300 μL PBS suspension. TSA plates were analyzed following a 48 h incubation at 35°C. BA plates were analyzed following a 48 h incubation at 35°C. R2A plates were analyzed following a seven-day incubation at 25°C. PDA plates were analyzed following a 7-day incubation at 30°C. Isolates were sub-cultured onto TSA plates and identified at the genus level using the Vitek 2 compact bacterial identification system (bioMérieux USA, St. Louis, MO) or Applied Biosystems Applied Biosystems 3500 Series Genetic Analyzer (Applied Biosystems, Waltham, MA) previously (Mazhari, 2021). Isolates were sub-cultured onto TSA plates and incubated for 24 h at 35°C. A 10 μL loopful of bacteria was transferred into a Microbank^®^ tube (Pro-Lab Diagnostics, Round Rock, TX), which contains a proprietary cryopreservation solution, vortexed for 30 s and stored at −80°C.

**Table 1:** Summary of hybrid genome assemblies.

Sample name	Curation lab	Equivalent ISO level^a^	Location	BioSample ID	No. contigs	Genome size (bp)	N50 (bp)	GC content (%)	Coverage (x)^b^	Genome Completion (%)
1813sda1	Cold	N/A	Glove box	SAMN29043843	2	5,662,393	5,204,374	34	77	100
2987tsa1	Cosmic Dust	5	Floor	SAMN29043850	1	4,088,722	4,088,622	46	92	99
1735sda2	Hayabusa	5	Floor	SAMN29043840	4	4,055,684	4,035,936	38	55	100
2090tsa1	Hayabusa	5	Table	SAMN29043852	7	5,745,698	5,311,671	34	88	99
1780r2a1	Lunar	6	Floor	SAMN29043839	1	4,184,516	4,184,416	38	108	99
1781tsa1	Lunar	6	Floor	SAMN29043842	2	7,080,870	7,075,035	45	26	99
2933tsa1	Lunar	6	Air	SAMN29043848	3	5,356,151	5,043,971	40	67	98
2941sda1	Lunar	6	Floor	SAMN29043849	6	6,929,459	6,868,407	49	17	99
1943r2a1	Lunar	6	Floor	SAMN29043844	1	3,749,596	3,749,496	41	77	100
2069sda1	Meteorite	7	Air	SAMN29043846	3	5,795,782	5,505,279	38	55	98
1370ba1	Meteorite	7	Floor	SAMN29043836	1	3,659,009	3,658,909	42	141	99
1480ba3	Meteorite	7	Floor	SAMN29043837	6	4,111,671	3,935,672	36	152	98
1663tsa1	Meteorite	7	Floor	SAMN29043838	38	5,550,983	614,346	36	82	100
2047tsa1	Meteorite	7	Table	SAMN29043845	3	3,909,693	3,692,152	39	124	99
1570r2a1	Cosmic Dust	5	Microscope	N/A^c^	6	10,188,618	5,353,115	35	54	100

a International Standards Organization (ISO) level (ISO 14644-1).

b Calculated for MiSeq reads only.

c Assembly removed because of contamination.

### 2.2 DNA isolation

For WGS, isolates were removed from the −80°C freezer, sub-cultured onto TSA plates and incubated at 35°C for 24 h. Isolates were sub-cultured after 24 h. to fresh plates. Individual colonies were then inoculated into 20 mL Hardy dx (cat no. Q85) TSB tubes. Tubes were incubated at 35°C while shaking at 200 rpm with loosened lids. After 24 h. liquid cultures were centrifuged at 10,000×*g* for 10 min. The cell pellet was resuspended in 1 mL sterile PBS and then centrifuged at 13,000×*g* for 2 min. to pellet the cells. The cell pellets were then resuspended in 480 μL of 50 mM (pH 8.0) ethylenediaminetetraacetic acid (EDTA) and 120 μL of 10 mg/mL egg white lysozyme, ultra-pure grade (Amresco, Solon, OH) was added to the resuspended cell pellet for cell lysis. Isolates were incubated at 37°C for 30–60 min. and centrifuged at 13,000×*g* for 2 min. The supernatant was removed and the Promega Wizard™ Genomic DNA Purification Kit was used for DNA extraction following the manufacturer’s protocol (Promega, Madison, WI). The Qubit 1X dsDNA High Sensitivity assay kit (Invitrogen, Waltham MA) was used to quantify DNA concentration from extracted DNA of samples. DNA size (~ 600 bp) and integrity was assessed with 4,200 TapeStation System with the Genomic DNA ScreenTape assay (Agilent, Palo Alto, CA).

### 2.3 Hybrid whole genome sequencing and post processing

A MinION Mk1C from Oxford Nanopore Technologies (ONT, Oxford, UK) was used for long-read DNA sequencing using the Rapid Barcoding Sequencing (SQK-RBK004,RBK_9054_v2_revQ_14Aug2019) kit according to manufacturer’s protocol. Two sequencing runs were conducted consisting of 8 and 12 DNA samples (100–400 ng). Samples were fragmented and barcoded then suspended in AMPure XP beads for cleaning and concentration. DNA concentration was measured using the Qubit Fluorometric Quantitation 1x dsDNA High Sensitivity Kit (ThermoFisher) and then added to 1 μL of rapid adapter buffer (ONT). The library was loaded onto a R.9.4.1 flow cell (ONT) under the MinKNOW program version 4.2.4 (ONT)^1^ was used to control a sequencing run of 72 h. Sequence reads were base-called and demultiplexed using Guppy version 4.3.4 (ONT); the high accuracy configuration in Guppy was used with a minimum quality score of 7 and barcode score of 40.

An Illumina MiSeq was used for short-read DNA sequencing using the MiSeq Reagent Kit v3 with paired-end reads, following the Illumina DNA prep reference guide # 1000000025416 v09; 400 ng of high molecular weight DNA was used for library prep. Tagmentation, the process of cleaving templated DNA and addition of adapters, was followed according to the protocol provided by Illumina. DNA concentration was measured for 15 libraries using the Qubit Fluorometric Quantitation 1x dsDNA High Sensitivity Kit (ThermoFisher). Each library was normalized to 4 nM and pooled to 12 pM in a total volume of 180 μL. Denature and dilution instructions were followed according to MiSeq^®^ instrument protocol with libraries loaded onto MiSeq^®^ Reagent v3 cartridge. The MiSeq Reporter software version 2.6.2.3 was used to demultiplex and generate the fastq files for the Illumina short reads.

For hybrid genome processing, FastQC v0.11.9 (Andrews, 2010) and MultiQC v1.10.1 (Ewels et al., 2016) were used to assess sequence quality of both Illumina short reads and Nanopore long reads. The Bioinformatics platform EDGE Bioinformatics (Li et al., 2016) was used to analyze and assemble the raw sequence data using the hybrid whole genome pipeline. Reads were trimmed at a quality level of 30. The minimum sequence length was set to 50. The “N” base cut off was set to 10. Reads with adapters or contamination sequences were trimmed using Porechop (Wick et al., 2017a) and homopolymers greater than 15 poly A were removed. *De novo* assembly was done using Unicycler v0.4.9. (Wick et al., 2017b) at a minimum contig length of 200 bp and a minimum of 2000 reads. Miniasm (Li, 2018) was used to find consensus sequences at a minimum of 3. Reads were mapped with bowtie2 v2.4.3 (Langmead et al., 2018) at a max clip (number of clipped read characters) of 50 and a min mapq (mapping quality score) of 42. Contig taxonomy was classified with BLAST (Camacho et al., 2009). The assembled draft genomes were submitted for annotation using NCBI Prokaryotic Genome Annotation Pipeline (PGAP). The closest bacterial strain identity was determined using the Type (Strain) Genome Server (TYGS) (© 2016–2022 Leibniz Institute DSMZ) by comparing all genomes with available strain genomes in TYGS database and by extracting the 16 s rRNA sequence and aligning against the TYGS database. Genome quality and completeness was assessed using CheckM version 1.0.18 (Parks et al., 2015). A pairwise similarity of amino acids was generated with EzAAI (Kim et al., 2021) with default parameters. A16S rRNA maximum likelihood phylogenetic tree was constructed using ETE3 3.1.2 (Huerta-Cepas et al., 2016) aligned using MAFFT v6.861b on the GenomeNet^2^ and inferred using RAxML v8.2.11 on34 model GTRGAMMA with default parameters (Stamatakis, 2014). An estimated maximum likelihood phylogenomic tree using 119 single copy genes (SCGs) from the phylum Bacillota was created using GToTree (Lee, 2019) under default parameters. Reference sequences were found using the tool Megablast in NCBI. Phylogenomic tree was visualized in iTOL interactive tree of life (Letunic and Bork, 2021).

### 2.4 MALDI-TOF MS

Isolates were cultured for 24 hours at 35°C on tryptic soy agar (TSA) plates. Isolates were prepared for analysis by ethanol treatment and formic acid extractions as previously described (Freiwald and Sauer, 2009). Briefly, 300 μL of Ultra-Pure Water, High Performance Liquid Chromatography Mass Spectrometer (HPLC/MS) Grade 11, was added to each 1.5 mL microfuge tube (Eppendorf, Hamburg, Germany). A 10 μL loop of a single colony was inoculated from the plates into separate microfuge tubes for each isolate and vortexed until homogeneous before adding 900 μL of ethanol, 100% HPLC/MS Grade 12, and then vortexing for 30 s. Tubes were stored at 4°C and then centrifuged at 13,000×*g* speed for 2 min. to pellet the cells. The supernatant was decanted, and the cell pellet was centrifuged for another minute at 13,000×*g* before excess liquid was removed with a pipette and left to air dry. After 5 min. of drying, 50 μL of 70% HPLC/MS grade formic acid was added and tubes were vortexed for 30 s. and incubated for 5 min. at room temperature. An equal volume (50 μL) of 100% HPLC/MS Grade acetonitrile was added and the suspension was vortexed for 30 s. prior to being centrifuged at maximum speed (13,000×*g*) for 2 min. Taking care to avoid the pellet, 70 μL of supernatant was transferred to a fresh 1.5 mL microfuge tube and stored at −20°C. For analysis, 1 μL of supernatant was pipetted onto a MALDI steel target (Bruker p/n 8280800). Two size standards were prepared by applying 1 μL of bacterial test standard (BTS, Bruker p/n 8255343) solution and two spots were left blank. After the spots dried, 1 μL of matrix solution (HCCA matrix, Bruker) was added to each spot and the target was allowed to thoroughly air dry for 10 min. Targets were shipped overnight, with an ice pack, to the Proteomics and Mass Spectrometry Core Facility at Pennsylvania State University, (University Park, PA). Positive-ion mass spectra were acquired on a Bruker Ultraflextreme MALDI-TOF/TOF mass spectrometer as described previously (LaMontagne et al., 2021).

MALDI-TOF MS data was analyzed with a script modified from LaMontagne et al. (2021). Briefly, mass spectra were analyzed by cluster analysis using an R script that contains functions from MALDIquant (Gibb and Strimmer, 2012) for processing mass spectra. Pvclust (Suzuki and Shimodaira, 2006) was used to provide bootstrap probability values for clusters in the dendrogram. Philentropy (Drost, 2018) was used to calculate distance and similarity measures. iNEXT (Hsieh et al., 2016) was used for rarefaction analysis and ggplot2 (Wickham, 2016) was used to for biplots. RWeka (Hornik et al., 2009) was to test the coherence of MALDI-TOF taxonomic units (MTUs). This script first ran a preliminary cluster analysis to calculate cosine similarities between spectra generated from pairs of mass spectra calculate a signal-to-noise ratio (SNR) for mass spectra. Cosine similarities were calculated following (Strejcek et al., 2018) and a histogram of cosine similarities to define a threshold for MTUs. This threshold was set to a cosine similarity of 0.7. We then ran a loop that iteratively sampled random values for seven parameters (half-window for smoothing, baseline removal, half-window for alignment, alignment tolerance, SNR for alignment, half-window for peak detection, and peak detection SNR) that are used by MALDIquant to align spectra and detect peaks. This loop was run 1,200 times. Jaccard coefficients, calculated following (Starostin et al., 2015), were used as the optimization parameter. The output of the alignment loop was sorted by Jaccard coefficients and the set of parameters that gave the highest number of shared peaks were selected. A quality check was done by calculation of the SNR for the 10 largest peaks in each spectra and plotting these ratios against the number of peaks detected. Spectra with low SNRs (< 11) and few (< 15) peaks were removed after confirmation by visual inspection.

Significance of differences between pairwise similarities was calculated with an ANOVA, using base functions in R, and a Tukey test using the package agricolae (de Mendiburu and Yaseen, 2020). The level of alpha for the Tukey test was set to 0.0001. Agreement between partitioning of isolates was tested with the adjusted Wallace coefficient (Severiano et al., 2011), using an online tool.^3^ Partitions for MALDI-TOF data were defined by MTUs. Partitions for WGS were defined by species identifications obtained with the TYGS (see text footnote 3). Isolates that were not typed to the species level by WGS, but showed AAI values greater than 90%, were put in the same partition.

Raw mass spectra are available in Supplementary file S1 and the script used to process this data is available, in R markdown format, in Supplementary file S2. Input and output files to and from this script, including mass spectra and MALDI Biotyper^®^ scores, are available as Supplementary files S3–S6. Scripts used for the Tukey test are available in Supplementary file S7.

## 3 Results

The optimization loop showed a modal relationship between Jaccard coefficients and the number of peaks detected. The highest Jaccard coefficients were observed at 163 peaks (Supplementary file S8).

**Figure 1:**
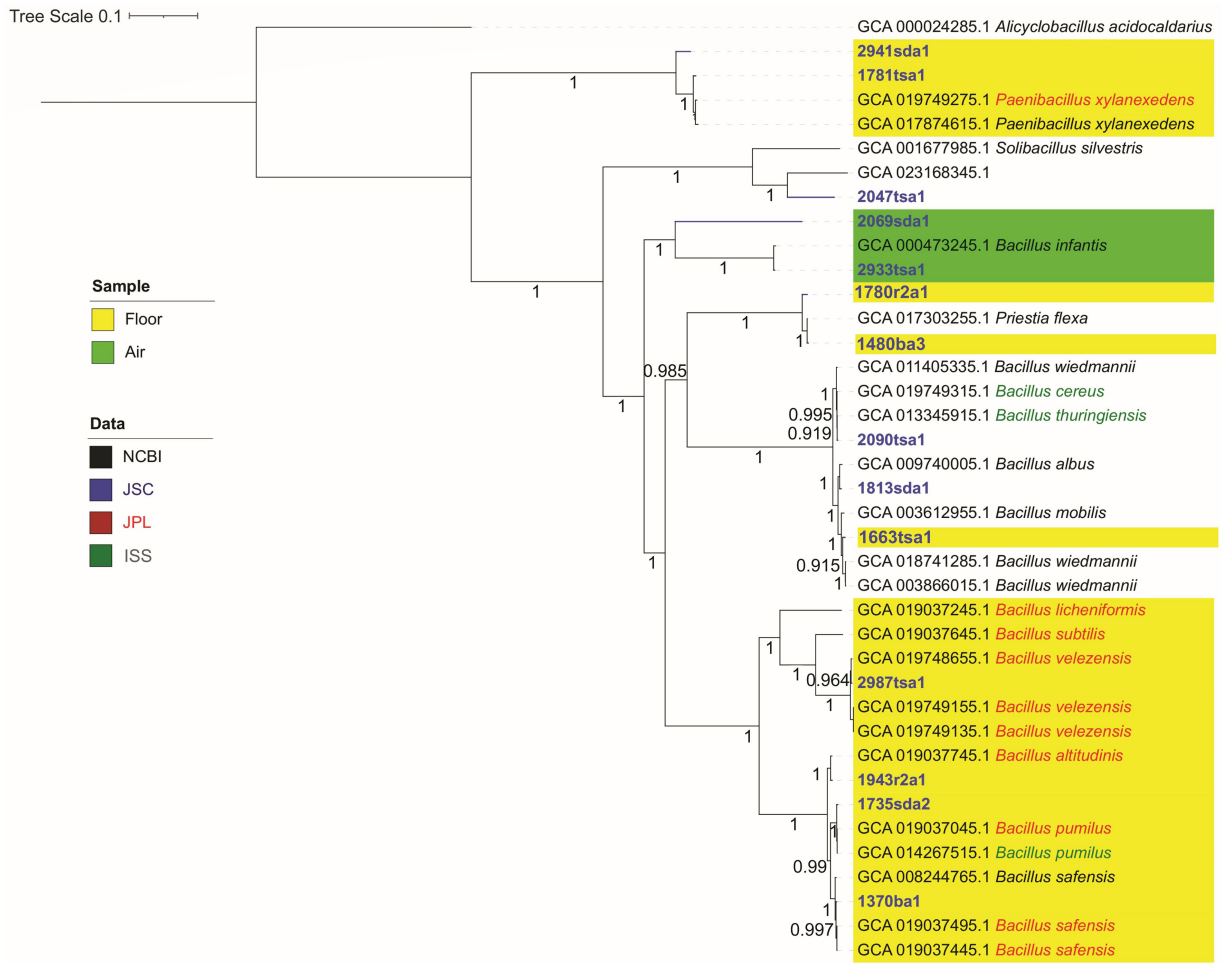
Genomic maximum likelihood tree showing phylogeny of isolates as determined by whole genome sequencing. Alignment and concatenation were based on amino acid sequences of single-copy core genes of the phylum firmicutes using GToTree and visualized using the iTOL interactive tree of life. Highlighted backgrounds indicate locations (floor or air) sampled in astromaterials cleanrooms. Other locations (not highlighted) are a glovebox (1813sda1), microscope (1570r2a1) and tables (2090tsa1 and 2047tsa1). Isolate name color indicates the origin of genome assemblies. Strain names for reference sequences are provided in Supplementary file S12.

From 15 isolates, 14 draft genomes were *de novo* assembled with greater than 98% mean completion (Table 1). GC contents for these isolates ranged from 34% to 49%, which is consistent with the nucleotide compositions typical for these genera (Lightfield et al., 2011) and mesophilic bacteria in general (Hu et al., 2022). Genome sizes ranged from 7.1 to 3.7 Mbp, which is typical for the phylum Bacillota (Martinez-Gutierrez and Aylward, 2022). Isolate 1781tsa1, which was classified as a *Paenibacillus* species (Figure 1), showed the largest genome size; isolate 1370ba1, which was classified as a strain of *Bacillus safensis* (Figure 1), showed the smallest genome size.

Bacterial identification by WGS generally agreed with identifications by the MALDI system at the genus level (Table 2); however, two isolates were classified as different genera. Isolate 1480ba3 was identified as *Priestia flexus* and isolate 2069sda1 was identified as *Peribacillus frigoritolerans* by WGS. These two isolates were both classified as *Bacillus* species by the MALDI system (Table 2). This reflects the recent assignment of *Bacillus flexus* to the novel genera *Priestia* (Gupta et al., 2020) and the proposal of *Peribacillus* as a novel genus (Patel and Gupta, 2020). Apparently, these changes in nomenclature have not been updated in the proprietary Bruker MALDI Biotyper^®^ database.

**Table 2:** Identification of bacterial isolates by DNA sequencing and MALDI-TOF MS.

Isolate	Edge^a^	MALDI Biotyper^®^ system	Biotyper^®^ score^b^	Whole genome sequencing
2090tsa1	3	*Bacillus cereus*	2.44	*Bacillus cereus*
1570r2a1	3	*Bacillus cereus*	2.28	*N.A.* ^c^
1813sda1	3	*Bacillus cereus*	2.32	*Bacillus* sp.
1663tsa1	3	*Bacillus cereus*	2.31	*Bacillus* sp.
1781tsa1	4	*P. amylolyticus*	2.34	*Paenibacillus* sp.
2941sda1	4	*Paenibacillus* sp.	1.96	*P. polysaccharolyticus*
1480ba3	5	*Bacillus flexus*	2.38	*Priestia flexa*
1780r2a1	5	*Bacillus flexus*	2.32	*Bacillus* sp.
1735sda2	6	*Bacillus pumilus*	2.06	*Bacillus* sp.
1943r2a1	6	*Bacillus altitudinis*	2.00	*Bacillus altitudinis*
1370ba1	7	*Bacillus safensis*	2.03	*Bacillus safensis*
2069sda1	8	*Bacillus simplex*	2.25	*Peribacillus frigoritolerans*
2047tsa1	8	*Lysinibacillus* sp.	1.83	*Lysinibacillus endophyticus*
2987tsa1	11	*B. amyloliquefaciens*	2.05	*Bacillus velezensis*
2933tsa1	12	*Bacillus infantis*	2.35	*Bacillus infantis*

a Nodes defined in Figure 2.

b Top score from MALDI Biotyper^®^ system. According to the manufacturer, a score > 1.8 is “probable genus identification,” > 2.0 is “secure species identification” and > 2.2 is “highly probably species identification.”

c Assembly removed because of contamination.

The MALDI system and WGS identified 13/15 and 9/14 at the species level, respectively, (Table 2). For 9 isolates identified at the species level by WGS, only 4 identifications were shared with identifications by MALDI-TOF MS. For example, isolate 2987tsa1 was identified as *B. amyloliquefaciens* and *B. velezensis* and isolate 2069sda1 was identified as *B. simplex* and *Peribacillus frigoritolerans* by MALDI-TOF MS and WGS and respectively (Table 2). In contrast, 16S rRNA gene taxonomy did not identify any of the isolates at the species level (Mazhari, 2021).

The topology of a phylogenomic tree generated from multiple single-copy core genes appeared largely coherent in terms of isolate identifications (Figure 1). That is, isolates generally formed monophyletic clades with representatives of their genera and the topology of this tree received strong bootstrap support (0.915–1.00). In contrast, a tree generated from 16S rRNA gene sequences (Supplementary file S9) showed several clades with relatively weak bootstrap support (0.24–0.41). Also, clades containing reference genomes for *Bacillus wiedmannii* were polyphylogenetic. This species clustered with two different *Bacillus* clades. Isolates did not appear to cluster by sample location.

Several clades in the phylogenomic tree showed that bacteria isolated from cleanrooms at JSC appeared closely related to bacteria isolated from cleanrooms at JPL, or similar built environments, like the International Space Station (ISS). For example, isolates identified as *Paenibacillus* species (2941sda1 and 1781tsa1), isolated from the floor of the Lunar curation lab at JSC, clustered with two strains *Paenibacillus xylanexedens* including a strain (PL-73) isolated from an air sample collected in a cleanroom at the JPL (GCA_019749275). Similarly, isolate 2090tsa1, which was isolated from a table in the Hayabusa lab at JSC, showed similarity to a strain (I1-R4) of *Bacillus cereus* isolated from an air sample collected at JPL. Further, *Bacillus* species (2987tsa1, 1943r2a1, 1735sda2, and 1370ba1) isolated from swabs of the cleanroom floors from the JSC Cosmic Dust, Lunar, Hayabusa, and Meteorite labs respectively, clustered with seven *Bacillus* strains isolated from spacecraft assembly cleanrooms at JPL and the ISS with strong bootstrap support (> 0.95, Figure 1).

The topology of a dendogram generated by mass spectra appeared coherent for isolates that were reliably identified at the species level by the MALDI system (Figure 2). In other words, representatives of the same species clustered together with strong bootstrap support (Figure 2). These clusters also appeared in the phylogenomic tree generated by WGS (Figure 1). In particular, edge 3 contained four isolates that classified as *Bacillus cereus* (Figure 2, Table 2). This clade corresponded to a clade, generated by WGS, that contained *B. cereus* and related species (Figure 1). Similarly, edge 5 contained two isolates that were classified as *Bacillus flexus* (Figure 2, Table 2). This clade corresponded to a clade, generated by WGS, that contained *Priestia flexa* and related species (Figure 1). A clade (edge 4) that contained two isolates that were classified as *Paenibacillus* species also received strong bootstrap support. Clades containing singletons generally appeared unreliable; however, edge 6 contained two *Bacillus* species that appeared closely related to each other in terms of mass spectra (Figure 2) and WGS (Figure 1).

**Figure 2:**
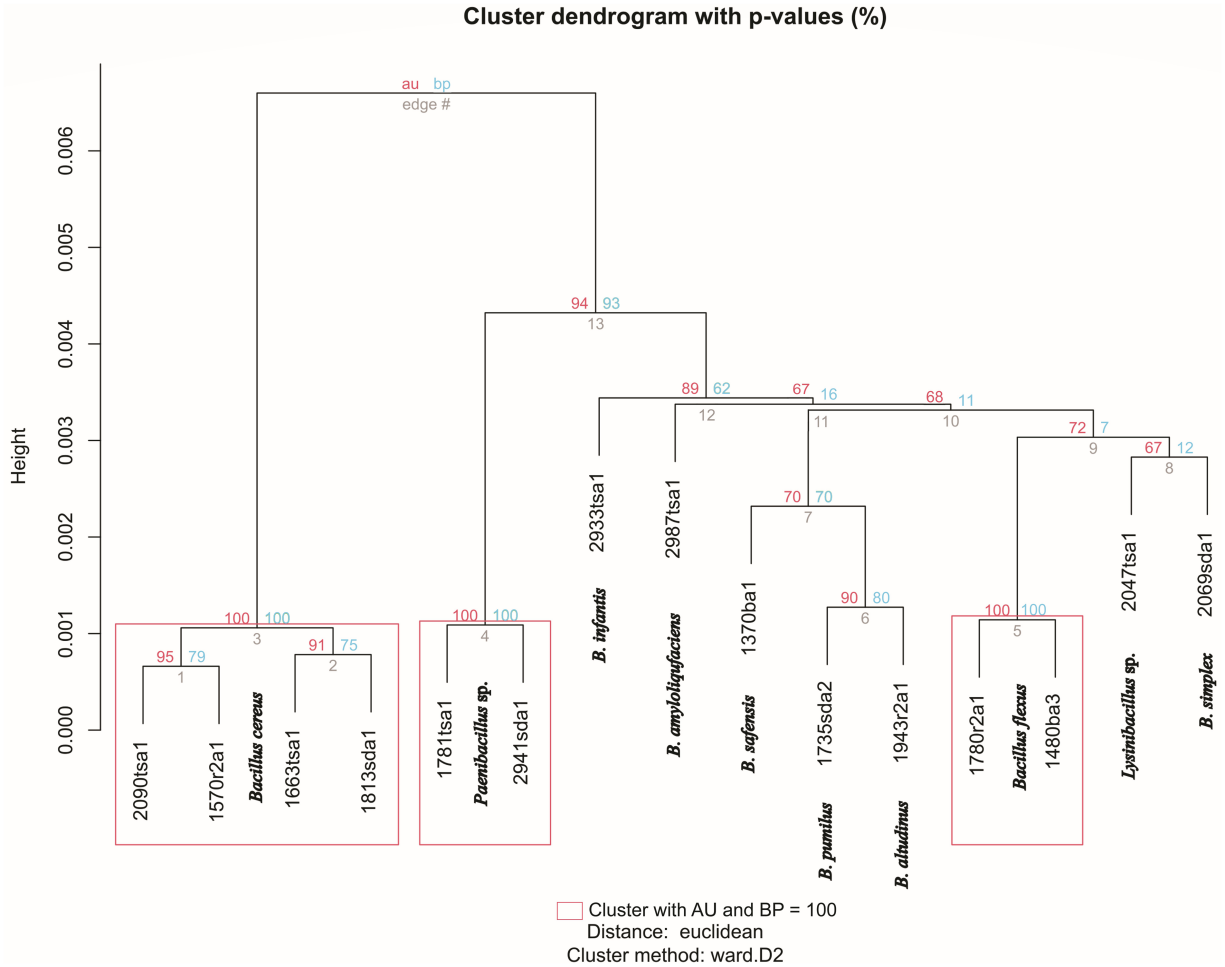
Hierarchical cluster dendrogram showing clustering of isolates based on mass spectra generated by MALDI-TOF. Approximately unbiased (AU, red values) and bootstrap probabilities (BP, blue values) represent *p*-values assigned by pvclust. Species identification, as assigned by Bruker MALDI Biotyper system is indicated at each node. Height indicates the degree of similarity between strains. Rectangles indicate clusters with AU and BP values of 100%.

Partitioning of isolates into MTUs, by MALDI-TOF, generally agreed with partitions defined by WGS. The adjusted Rand coefficient, for congruence of microbial typing method, was 0.95 and the adjusted Wallace coefficient was 0.90, with a 95% confidence interval of 0.80 to 1.00.

**Figure 3:**
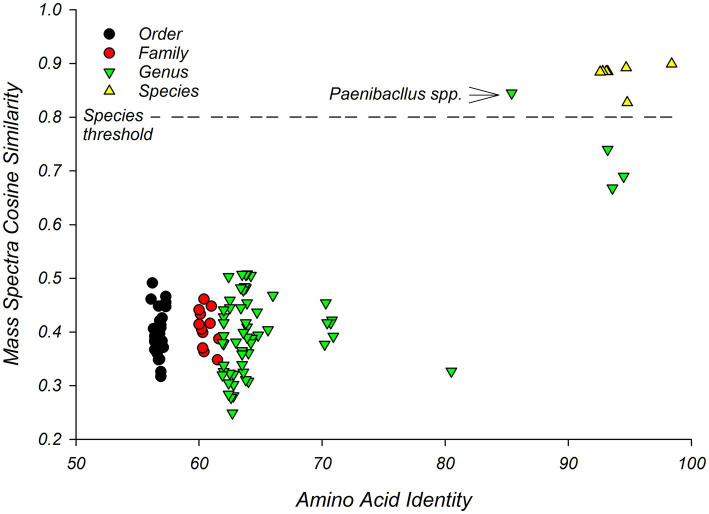
Pairwise similarity graph of MALDI-TOF and whole genome sequencing showing threshold for defining *Bacillus* species. Comparison of similarity between strains as assessed by MALDI-TOF MS and WGS shows that pairs of strains of the same *Bacillus* species showed cosine similarities of mass spectra of greater than 0.8. This threshold is indicated with a dashed line. X-axis presents amino acid identity between pairs of isolates. Y-axis presents pairwise similarity coefficients calculated from mass spectra. The comparison between the two *Paenibacillus* species is indicated.

Pairs of isolates that were classified as members of the same species by the MALDI system, showed high similarity to each other in terms of WGS (Figure 3). This supports the hypothesis that MALDI-TOF MS and WGS are comparable. Specifically, pairwise similarity of the amino acid sequences from single-copy core genes, predicted from the draft genomes, ranged from 93% to 98% AAI for pairs of isolates from the same species. These pairs showed cosine similarities, generated from mass spectra, that ranged from 0.83 to 0.90 with an average of 0.88 (Figure 3). Pairwise comparisons of mass spectra between members of the same species were significantly (*p* = 0.0001) larger than pairwise comparisons for members of different species (Figure 3, Supplementary file S7).

Pairwise similarities of mass spectra at the genus level were on average lower (0.42); however, the pair of *Paenibacillus* species showed a high similarity to each other in terms of cosine similarity of mass spectra (0.85) but a relatively low AAI (Figure 3). Similarly, three *Bacillus* isolates (1370sda2, 1735sda2, and 1943r2a1), which were classified as different species by both WGS sequencing and mass spectra (Table 2), showed relatively high AAI similarities (93–95%). These three isolates showed cosine similarities of 0.68 to 0.74, which were lower than expected given trends in Figure 3.

Pairwise comparisons of isolates that were assigned to different families (Paenibacillaceae and Bacillaceae) of the order Bacillales, and different genera (*Lysinibacillus* and *Bacillus*) of the family Bacillaceae, were resolved by AAIs but not by cosine similarities (Figure 3). These comparisons at the order and family level averaged 57 (56–57)% and 61 (60–62)%, respectively, for AAIs and 0.40 (0.33–0.47), 0.41 (0.35–0.46) respectively for cosine similarities (Supplementary file S7).

At a cosine similarity of 0.8, which appeared to resolve genera and species of *Bacillus* (Figure 3), MALDI-TOF MS detected 10 MALDI-TOF taxonomic units (MTUs) in a library of 15 isolates. This library contained three MTUs with more than one isolate and seven singletons. This corresponds to a coverage of 59%; extrapolation to a library of 28 isolates would give 80% coverage (Supplementary file S10).

## 4 Discussion

MALDI-TOF showed a resolution comparable to that of WGS. Both MALDI-TOF MS and WGS identified *Bacillus* strains consistently at the genus level (Table 2) and the relationship between isolates as assessed by phylogenomic analysis (Figure 1) agreed with clustering generated by mass spectra (Figure 2). Comparison of the similarity between isolates as assessed by WGS, suggested a threshold for identifying species of *Bacillus* isolates by MALDI-TOF MS. Pairs of strains that showed cosine similarities of mass spectra of >0.80, were reliably identified at the species level by the MALDI system and showed AAI, as assessed by WGS, of >92% (Figure 3). The high cosine similarities for comparisons within *Bacillus* species relative to comparison between *Bacillu*s species (Figure 3) confirms that MALDI-TOF MS provides an accurate representation of species diversity and can differentiate between related *Bacillus* species (Celandroni et al., 2016; Hotta et al., 2011; Lasch et al., 2009). This suggests MALDI-TOF provides a quick, cost-effective, and accurate method of identifying microbes that contaminate astromaterials curation facilities.

This threshold for defining a species by MALDI-TOF MS agrees with the cosine similarity threshold of 0.79 set by Strejcek et al. (2018). This proposed threshold of cosine similarity threshold (0.80) could result in the misclassification of *Paenibacillus* strains, which appear to be different species as assessed by AAI (Figure 3). AAI levels of >92%, observed for within-species comparisons is lower than the widely used threshold for defining a species for prokaryotes by AAI of 95% (Konstantinidis and Tiedje, 2005); however, this higher threshold for AAI levels may be biased towards clinical isolates (Rosselló-Mora, 2005).

Phylogenomic analysis showed several isolates clustered with *Bacillus* spp. isolated from other studies of cleanrooms and the ISS (Figure 1). This suggests there is a cosmopolitan class of *Bacillus* strains associated with cleanrooms and similar built environments, including the ISS (Blaustein et al., 2019). Microbes in the ISS and cleanrooms face similar selective pressures, including low nutrient availability and humidity. These “extreme environments” (Checinska Sielaff et al., 2019; La Duc et al., 2003) may share many microbes; however, this biogeographical pattern was not consistent. For example, the isolate identified as *Lysinbacillus endophyticus* (2047tsa1) clustered with a *Lysinbacillus* species isolated from the ant microbiome and the isolate that classified as *Bacillus infantis* (2933tsa1) clustered with a *Bacillus* species isolated from a marine system. Clearly, these species are not exclusively observed in cleanrooms; however, their detection can be explained. *Lysinibacillus* species have been isolated from air samples (Wong et al., 2019) and novel species, that are closely related to *Bacillus infantis,* have been isolated from cleanrooms (Seiler et al., 2013).

For routine monitoring, this proteomics approach could replace Vitek2 and 16S rRNA sequencing, which are widely used in environmental monitoring programs. The resolution of MALDI-TOF MS could also help investigators prioritize isolates for deeper analysis by WGS and biochemical tests. For example, genetic and functional characteristics of MTUs could be used to identify novel species with unique metabolic capabilities. The high resolution of MALDI-TOF also allows for tracking the source of microbial contamination, as has been applied to aquatic systems (Giebel et al., 2008; Siegrist et al., 2007).

### 4.1 Limitations

Sample size limits broader interpretation of the above results. Only 15 isolates were selected for this study. This corresponds to a coverage of a little more than half of the MTUs in the system sampled (Supplementary file S10). Accordingly, many of the isolates in the library were singletons (7/15), so in terms of their mass spectra we have little to compare them to internally. Despite the small sample size, precision of measurements provided the power to compare MALDI-TOF MS and WGS. Specifically, a Tukey test run at an alpha of 0.0001 showed that similarities of mass spectra generated by MALDI-TOF was significantly higher for comparisons within species than comparisons between species (Supplementary file S7).

The topology of the dendrogram generated from mass spectra (Figure 2) did not appear coherent for isolates that were not closely related, in terms of AAI or mass spectra. This may reflect the low similarity of mass spectra for isolates that are not members of the same species (Figure 3) and is consistent with previous studies that showed that dendrograms generated by hierarchical clustering of mass spectra do not consistently follow phylogenetically coherent topologies for *Bacillus* (Lasch et al., 2009) and *Lactobacillus* species (Douillard et al., 2013).

Lack of reference spectra can limit the ability of the MALDI-TOF system to identify bacteria isolated from built and natural environments (Popović et al., 2017). This can be addressed by developing internal databases, which has been initiated for *Bacillus* species isolated from cleanrooms (da Costa et al., 2022) and spacecraft assembly facilities (Seuylemezian et al., 2018). These custom databases improve with the addition of mass spectra from multiple strains. For example, Erler et al. (2015) generated a custom database for identifying *Vibrio* species isolated from aquatic environments; this expanded database of 997 mass spectra dramatically improved bacterial identification by MALDI-TOF MS.

## 5 Summary

MALDI-TOF MS can efficiently identify species of the genus *Bacillus* that are frequently isolated from facilities where astromaterials are curated, and similar built environments, with a resolution comparable to WGS. Implementation of this proteomics approach would require development of database of mass spectra.

## Data Availability

WGS data is available at NCBI BioProject PRJNA849219. Mass spectra generated by MALDI-TOF MS are available in Supplementary file S1. Scripts used to process this data are available in html format as Supplementary file S2. Other raw data used in these scripts are available in Supplementary files S3–S6. Processed mass spectra are available in mzML format (Deutsch, 2010) in Supplementary file S11. Genes used in calculation of AAI are available in Supplementary file S12.

## Footnotes

^1^ https://community.nanoporetech.com/docs/prepare/library_prep_protocols/experiment-companion-minknow/v/mke_1013_v1_revdc_11apr2016

^2^ https://www.genome.jp/tools/ete/

^3^ http://www.comparingpartitions.info/
