# Supplementary material for: Resolution of MALDI-TOF compared to whole genome sequencing for identification of Bacillus species isolated from cleanrooms at NASA Johnson Space Center
Source: Front Microbiol. 2025 Apr 9;16:1499516. doi: 10.3389/fmicb.2025.1499516 (PMC12017291; doi:10.3389/fmicb.2025.1499516)
Supplement: Supplementary file 3 [file Data_Sheet_3.pdf]

jscSum

Michael G. LaMontagne

10/31/2023

Load packages

```
library("MALDIquant")
```

```
##
## This is MALDIquant version 1.22.1
## Quantitative Analysis of Mass Spectrometry Data
##  See '?MALDIquant' for more information about this package.
```

```
library("MALDIquantForeign")
library("pvclust")
library(philentropy)
library(ggplot2)
library(iNEXT)
library(RWeka)

packageVersion("MALDIquant")
```

```
## [1] '1.22.1'
```

```
packageVersion("MALDIquantForeign")
```

```
## [1] '0.13'
```

```
packageVersion("pvclust")
```

```
## [1] '2.2.0'
```

```
packageVersion("philentropy")
```

```
## [1] '0.7.0'
```

```
packageVersion("iNEXT")
```

```
## [1] '3.0.0'
```

```
packageVersion("ggplot2")
```

```
## [1] '3.4.2'
```

```r
packageVersion("RWeka")
```

```
## [1] '0.4.46'
```

```r
# write record of packages and versions
sessionInfo()
```

```
## R version 4.3.0 (2023-04-21 ucrt)
## Platform: x86_64-w64-mingw32/x64 (64-bit)
## Running under: Windows 11 x64 (build 22621)
##
## Matrix products: default
##
##
## locale:
## [1] LC_COLLATE=English_United States.utf8
## [2] LC_CTYPE=English_United States.utf8
## [3] LC_MONETARY=English_United States.utf8
## [4] LC_NUMERIC=C
## [5] LC_TIME=English_United States.utf8
##
## time zone: America/Chicago
## tzcode source: internal
##
## attached base packages:
## [1] stats     graphics  grDevices utils     datasets  methods   base
##
## other attached packages:
## [1] RWeka_0.4-46           iNEXT_3.0.0            ggplot2_3.4.2
## [4] philentropy_0.7.0      pvclust_2.2-0          MALDIquantForeign_0.13
## [7] MALDIquant_1.22.1
##
## loaded via a namespace (and not attached):
##  [1] sass_0.4.5              utf8_1.2.3              generics_0.1.3
##  [4] stringi_1.7.12          digest_0.6.31           magrittr_2.0.3
##  [7] evaluate_0.20           grid_4.3.0              fastmap_1.1.1
## [10] plyr_1.8.8              jsonlite_1.8.4          fansi_1.0.4
## [13] scales_1.2.1            XML_3.99-0.14           jquerylib_0.1.4
## [16] RWekajars_3.9.3-2       cli_3.6.1               rlang_1.1.1
## [19] munsell_0.5.0           base64enc_0.1-3         withr_2.5.0
## [22] cachem_1.0.8            yaml_2.3.7              tools_4.3.0
## [25] parallel_4.3.0          reshape2_1.4.4          dplyr_1.1.2
## [28] colorspace_2.1-0        vctrs_0.6.2             readBrukerFlexData_1.9.1
## [31] R6_2.5.1                lifecycle_1.0.3         stringr_1.5.0
## [34] pkgconfig_2.0.3         rJava_1.0-6             readMzXmlData_2.8.2
## [37] pillar_1.9.0            bslib_0.4.2             gtable_0.3.3
## [40] glue_1.6.2              Rcpp_1.0.10             xfun_0.39
## [43] tibble_3.2.1            tidyselect_1.2.0        rstudioapi_0.14
## [46] knitr_1.42              htmltools_0.5.5         rmarkdown_2.21
## [49] compiler_4.3.0
```

Import spectra and names

```
spectraQ <- readRDS("spectraJSCqWGS.Rds")
speciesNamesQ <- as.data.frame(read.csv("speciesNamesQ.csv"))
head(speciesNamesQ)
```

```
##    aaiCode spot
## 1     BTSa   A1
## 2     BTSa   A2
## 3     BTSa  H11
## 4     BTSa  H12
## 5 j1370ba1   D1
## 6 j1370ba1   D2
```

set alignment parameters

```
hwSmth <- 70
rmvbI <- 100
hwA <-  50
snrA <-  2.2
tolA <- 0.029
hwD <- 70
snrD <- 6.4
```

create reference peaks

```
rR <- createMassPeaks(mass=c(2833, 4362, 5095, 5379, 5392, 5396, 6254,
                             6268, 6278, 6291, 6314, 6328, 6353, 6410, 7272, 9533, 9552),
                      intensity=rep(1, 17))
rR
```

```
## S4 class type            : MassPeaks
## Number of m/z values     : 17
## Range of m/z values      : 2833 - 9552
## Range of intensity values: 1e+00 - 1e+00
## Range of snr values      : NA - NA
## Memory usage             : 1.531 KiB
```

align spectra

```
spectra <- smoothIntensity(spectraQ, method="SavitzkyGolay", halfWindowSize=hwSmth)
spectra <- removeBaseline(spectra, method="SNIP",iterations=rmvbI)
spectraT <- calibrateIntensity(spectra, method="TIC")
spectra <- (alignSpectra
               (spectraT, halfWindowSize=hwA, SNR=snrA, reference = rR,
                 tolerance=tolA, warpingMethod="lowess"))
#if ('try-error' %in% class(spectra)) next
##---metadata
LB  <- length(spectra)
LB
```

```
## [1] 135
```

detect peaks and plot dendogram

```
metaData(spectra[[LB]])$sampleName
```

```
## [1] "d2069_SDA_1"
```

```
spots <- sapply(spectra, function(x)metaData(x)$spot)
#species <- sapply(spectra, function(x)metaData(x)$sampleName)
species <- speciesNamesQ$aaiCode
avgSpectra <-
  averageMassSpectra((spectra), labels=paste0(species))
## detectPeaks
peaks <- detectPeaks(avgSpectra, SNR=snrD, halfWindowSize=hwD)
##--export peaks
peaks <- binPeaks(peaks)
spots <- sapply(avgSpectra, function(x)metaData(x)$spot)
#species <- sapply(avgSpectra, function(x)metaData(x)$sampleName)
species <- labels(spots)
species <- factor(species)
n <- length(avgSpectra)
featureMatrix <- intensityMatrix(peaks, avgSpectra)
rownames(featureMatrix) <- paste(species)
peaksNew <- intensityMatrix(peaks)
rownames(peaksNew) <- paste(species)
##--write matrix
FMtrnsp <- t(featureMatrix)
nrow(FMtrnsp)
```

```
## [1] 142
```

```
# calculate similarity matrix
x <- cor(FMtrnsp)
rownames(x) <- paste(species)
# calcualte cos simmilarity
simCos <- coop::cosine(FMtrnsp)
rownames(simCos) <- paste(species)
## calculate Jaccard coefficients
N <- intensityMatrix(peaks)
rownames(N) <- paste(species)
N[N>0] <- 1
N[is.na(N)] <- 0
k <- distance((N), method = "jaccard")
```

```
## Metric: 'jaccard'; comparing: 17 vectors.
```

```
rownames(k) <- paste(species)
Rich <- ncol(N)
richE <- sum(N[1,])
Rich
```

```
## [1] 142
```

plot dendogram

```
pv <- pvclust(FMtrnsp,
              method.hclust="ward.D2",
              method.dist="euclidean", nboot=1000)
```

```
## Bootstrap (r = 0.5)... Done.
## Bootstrap (r = 0.6)... Done.
## Bootstrap (r = 0.7)... Done.
## Bootstrap (r = 0.8)... Done.
## Bootstrap (r = 0.89)... Done.
## Bootstrap (r = 1.0)... Done.
## Bootstrap (r = 1.1)... Done.
## Bootstrap (r = 1.2)... Done.
## Bootstrap (r = 1.3)... Done.
## Bootstrap (r = 1.39)... Done.
```

```
plot(pv, print.num=FALSE)
```

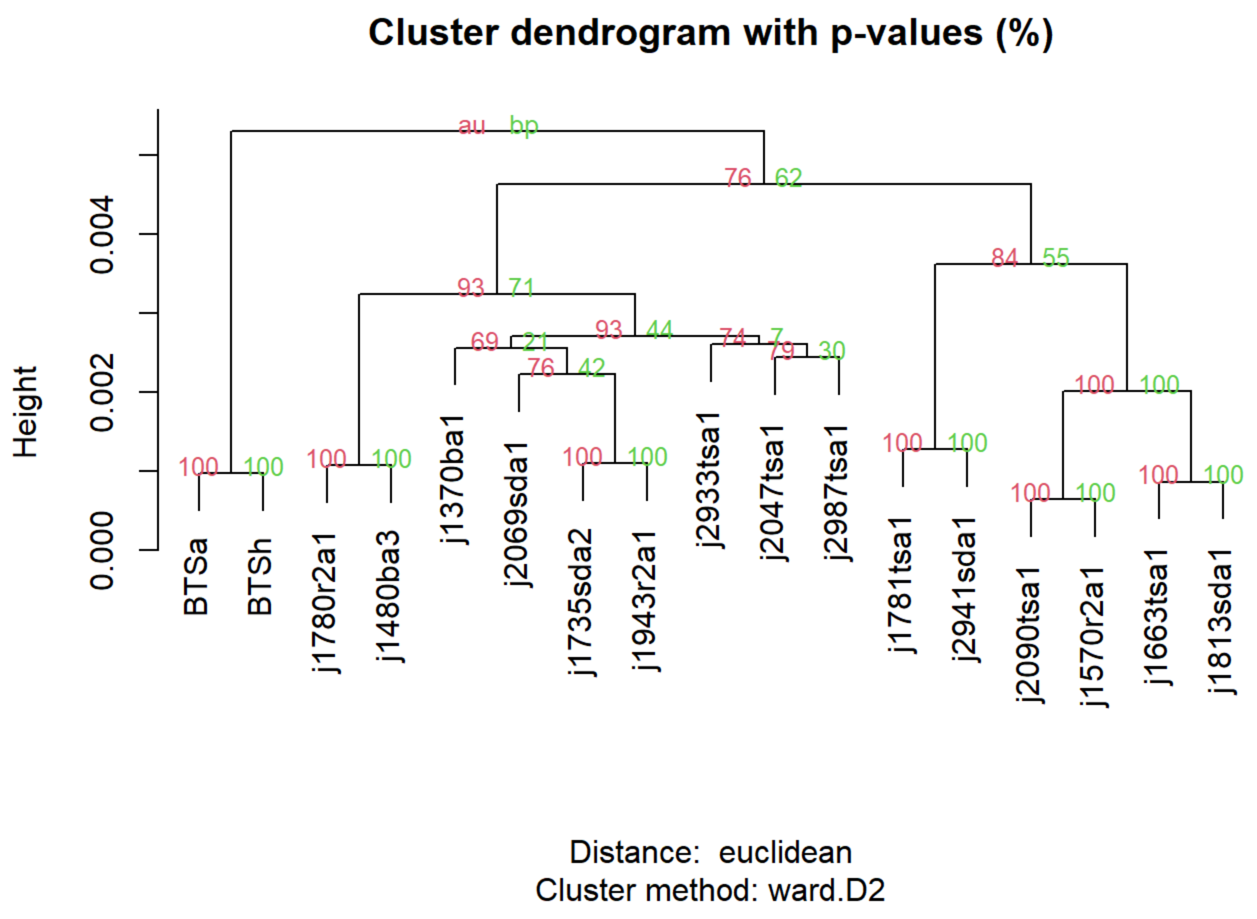

export mzML

```
#exportMzML(avgSpectra, path="spectraJSC.mzML")
```

create dataframe of simCos pairwise comparisons

```
xySC <- t(combn(colnames(simCos), 2))
xyS <- data.frame(xySC, dist=simCos[xySC])
names(xyS)[3] <- "distS"
xyS$sPair <- paste(xyS$X1,xyS$X2,sep="-")
head(xyS)
```

```
##     X1        X2     distS          sPair
## 1 BTSa  j1370ba1 0.3766249  BTSa-j1370ba1
## 2 BTSa j1663tsa1 0.5189602 BTSa-j1663tsa1
## 3 BTSa j1735sda2 0.3503295 BTSa-j1735sda2
## 4 BTSa j1780r2a1 0.3550010 BTSa-j1780r2a1
## 5 BTSa j1781tsa1 0.4348768 BTSa-j1781tsa1
## 6 BTSa j1813sda1 0.4663108 BTSa-j1813sda1
```

```
#
Km <- 0.2
y0 <- 0
# weigh simCos with Jaccard
rownames(k) <- paste(species)
colnames(k) <- paste(species)
k2 <- 1 - k
kW <- y0 + (k2/(Km+k2))
# weigh matrix
cosW <- (simCos + kW)/2
# create dataframe of weighted pairwise comparisons
xyW <- t(combn(colnames(cosW), 2))
xyW <- data.frame(xyW, distCW=cosW[xySC])
names(xyW)[3] <- "distWref"
xyS <- data.frame(xyS, xyW[3])
head(xyS)
```

```
##     X1        X2     distS          sPair  distWref
## 1 BTSa  j1370ba1 0.3766249  BTSa-j1370ba1 0.3932305
## 2 BTSa j1663tsa1 0.5189602 BTSa-j1663tsa1 0.4838391
## 3 BTSa j1735sda2 0.3503295 BTSa-j1735sda2 0.2409542
## 4 BTSa j1780r2a1 0.3550010 BTSa-j1780r2a1 0.2684096
## 5 BTSa j1781tsa1 0.4348768 BTSa-j1781tsa1 0.3490174
## 6 BTSa j1813sda1 0.4663108 BTSa-j1813sda1 0.4575144
```

add dataframe of Jaccards

```
xyJC <- t(combn(colnames(k2), 2))
xyJ <- data.frame(xyJC, distJ=k2[xyJC])
head(xyJ)
```

```
##     X1        X2      distJ
## 1 BTSa  j1370ba1 0.13888889
## 2 BTSa j1663tsa1 0.16279070
## 3 BTSa j1735sda2 0.03030303
## 4 BTSa j1780r2a1 0.04444444
## 5 BTSa j1781tsa1 0.07142857
## 6 BTSa j1813sda1 0.16279070
```

```
## merge jaccard and simCos
xyJS <- data.frame(xyS,xyJ[3])
head(xyJS)
```

```
##     X1        X2     distS          sPair  distWref      distJ
## 1 BTSa  j1370ba1 0.3766249  BTSa-j1370ba1 0.3932305 0.13888889
## 2 BTSa j1663tsa1 0.5189602 BTSa-j1663tsa1 0.4838391 0.16279070
## 3 BTSa j1735sda2 0.3503295 BTSa-j1735sda2 0.2409542 0.03030303
## 4 BTSa j1780r2a1 0.3550010 BTSa-j1780r2a1 0.2684096 0.04444444
## 5 BTSa j1781tsa1 0.4348768 BTSa-j1781tsa1 0.3490174 0.07142857
## 6 BTSa j1813sda1 0.4663108 BTSa-j1813sda1 0.4575144 0.16279070
```

```
# predict simCos from Jaccard
xyJS$cosPred <- y0 + (xyJS$distJ/(Km+xyJS$distJ))
plot.default(xyJS$distJ, xyJS$distS, xlab = "Jaccard", ylab = "SimCos")
```

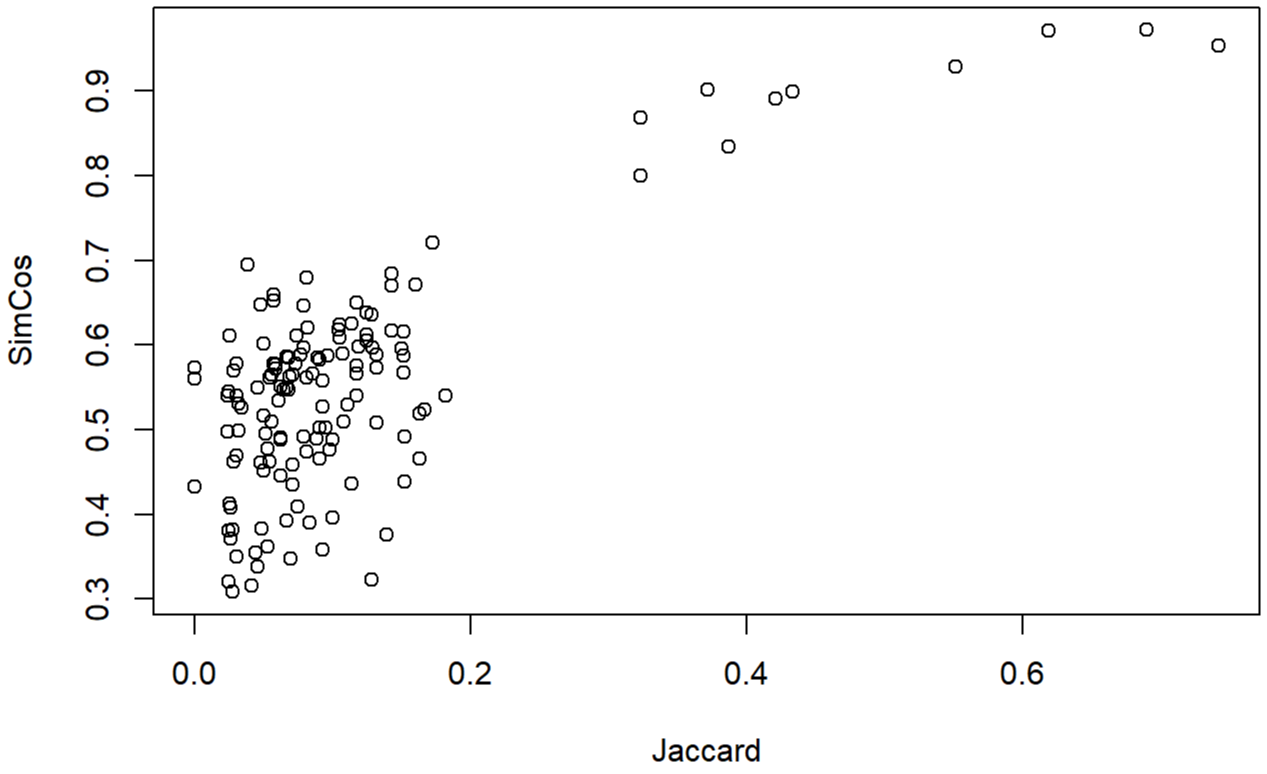

plot weighted

```
plot.default(xyJS$distJ, xyJS$distWref, xlab = "Jaccard", ylab = "SimCosW")
```

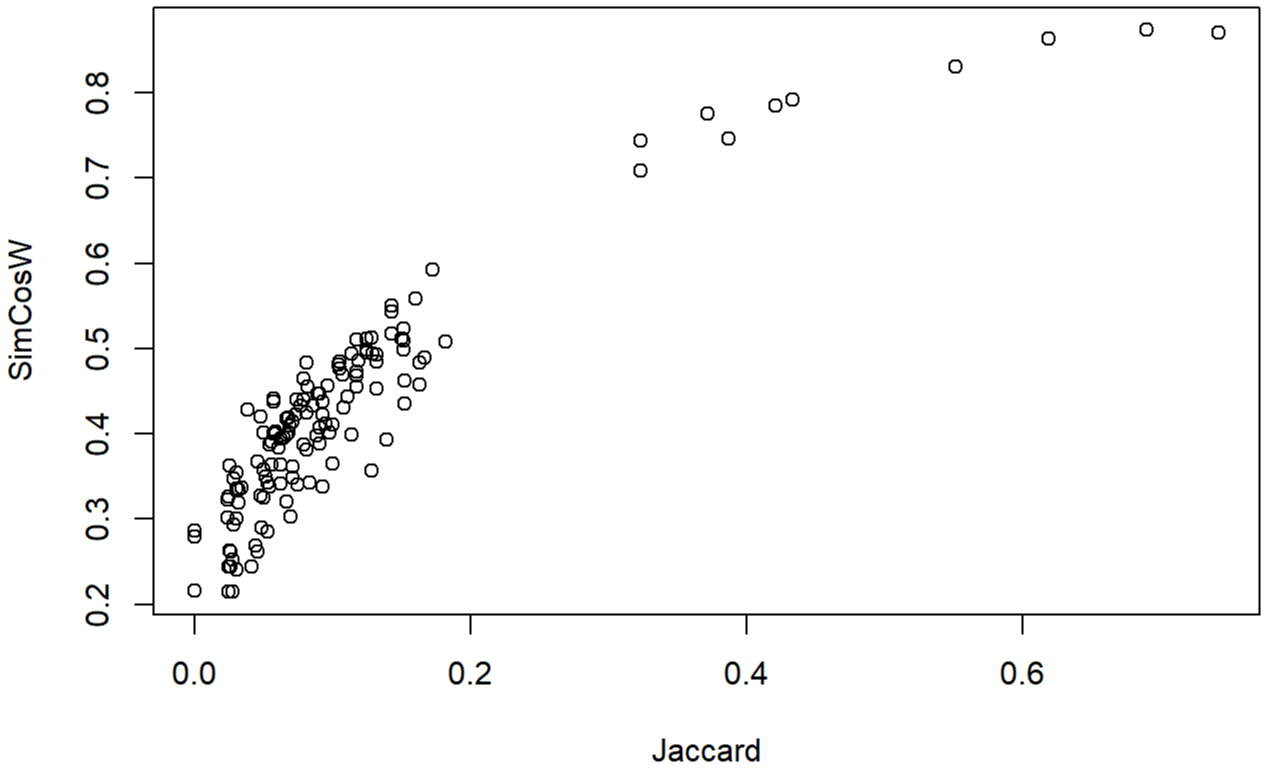

histogram of similarity coefficients

```
hist(xyJS$distWref)
```

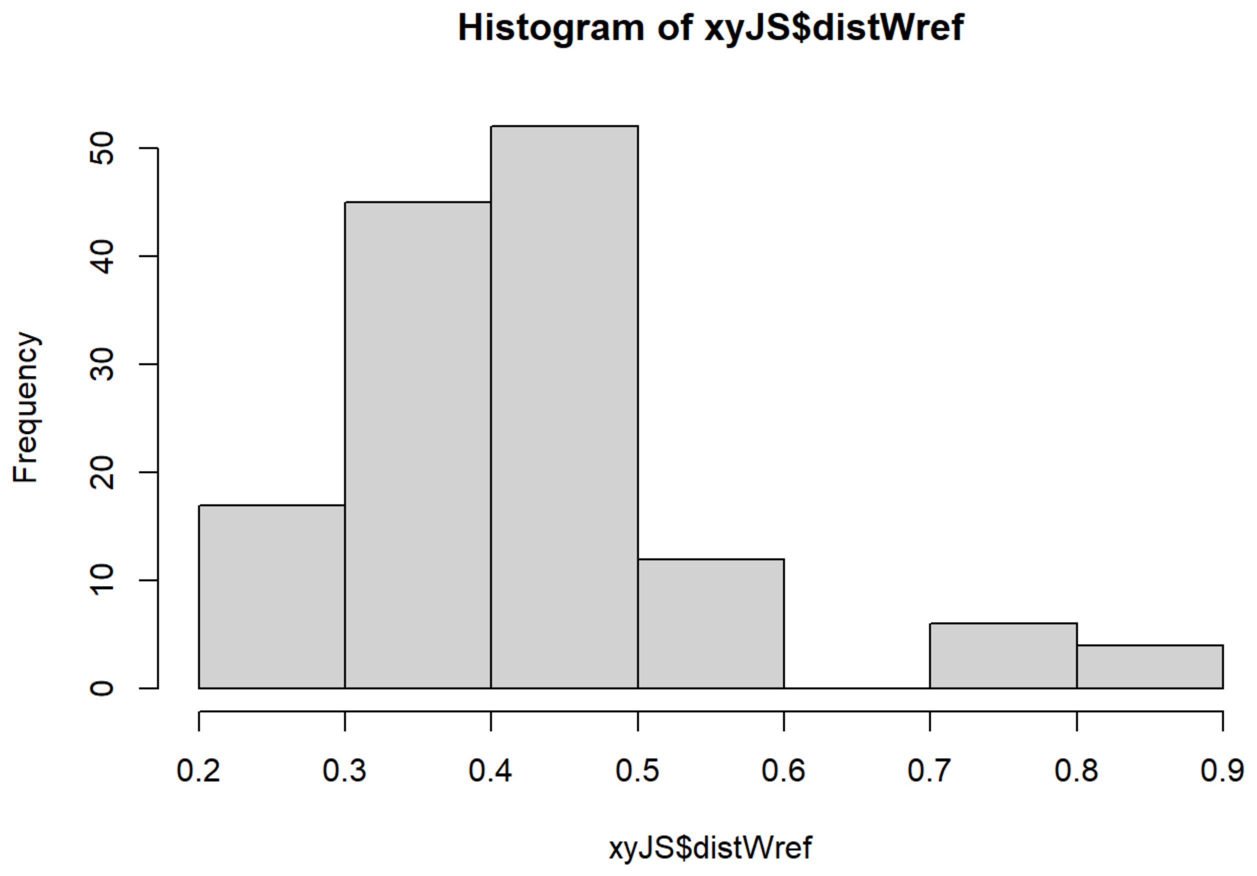


```
xyRef <- xyJS
write.csv(xyRef, "xyRef.csv")
```

reset variables

```
y <- c(0, 0, 0, 0, 0, 0, 0, 0, 0, 0, 0, 0, 0, 0, 0, 0, 0, 0, 0, 0, 0, 0)
Dq <- matrix(y, nrow=22, ncol=1)
rownames(Dq) <- c("hwSmth", "rmvbI", "hwAlgn", "snrAlgn", "tolAlgn", "hwDtct", "snrDtct", "peaks", "Cor12",
"RichE", "K12", "Pass", "MaxRep", "out16S", "fitTry","fit16W", "out16W", "Km", "y0", "distJ", "distS", "dis
tW")
# reset counts
try <- c(0)
Pass <- c(0)
M12 <- c(0)
```

align quality spectra to determine parameters that separate reference MTUs from unrelated isolates

```
BTS <- c(1:4, 41:44,97:100,110:111)
spectraJ <- spectraQ [-(BTS)]
length(spectraJ)
```

```
## [1] 121
```

align quality spectra to determine parameters that separate reference MTUs from

```
speciesNamesJ <- as.data.frame(read.csv("speciesNamesJ.csv"))
head(speciesNamesJ)
```

```
##     aaiCode spot
## 1  j1370ba1   D1
## 2  j1370ba1   D2
## 3  j1370ba1   D3
## 4 j1663tsa1  D11
## 5 j1663tsa1  D12
## 6 j1663tsa1   D9
```

```
#saveRDS(spectraJ, "spectraJSCjWGS.Rds")
```

align quality spectra to determine parameters that separate

reference MTUs from unrelated isolates

```
## create blank variable for BTS correlation
K12 <- 0
##align
repeat{
  hwSmth <- 10*(sample(2:9, 1))
  rmvbI <- 25*(sample(2:4, 1))
  hwA <-  10*(sample(2:9, 1))
  snrA <-  (sample(20:30, 1))/10
  tolA <- (sample(20:30, 1))/1000
  hwD <- 10*(sample(4:10, 1))
  snrD <- (sample(40:99, 1))/10
  ##align
  spectra <- smoothIntensity(spectraJ, method="SavitzkyGolay", halfWindowSize=hwSmth)
  spectra <- removeBaseline(spectra, method="SNIP",iterations=rmvbI)
  spectraT <- calibrateIntensity(spectra, method="TIC")
  spectra <- try(alignSpectra
                (spectraT, halfWindowSize=hwA, SNR=snrA, reference = rR,
                  tolerance=tolA, warpingMethod="lowess"), silent = FALSE)
  if ('try-error' %in% class(spectra)) next
  ##---metadata
  LB  <- length(spectra)
  metaData(spectra[[LB]])$sampleName
  spots <- sapply(spectra, function(x)metaData(x)$spot)
#  species <- sapply(spectra, function(x)metaData(x)$sampleName)
 species <-  speciesNamesJ$aaiCode
  avgSpectra <-
    averageMassSpectra((spectra), labels=paste0(species))
  ## detectPeaks
  try(peaks <- detectPeaks(avgSpectra, SNR=snrD, halfWindowSize=hwD), silent = FALSE)
  if ('try-error' %in% class(avgSpectra)) next
  ##--export peaks
  peaks <- binPeaks(peaks)
  spots <- sapply(avgSpectra, function(x)metaData(x)$spot)
#  species <- sapply(avgSpectra, function(x)metaData(x)$sampleName)
 species <- labels(spots)
  species <- factor(species)
  n <- length(avgSpectra)
  featureMatrix <- intensityMatrix(peaks, avgSpectra)
  rownames(featureMatrix) <- paste(species)
  peaksNew <- intensityMatrix(peaks)
  rownames(peaksNew) <- paste(species)
  ##--write matrix
  FMtrnsp <- t(featureMatrix)
  nrow(FMtrnsp)
  # calcualte cos simmilarity
  simCos <- coop::cosine(FMtrnsp)
  rownames(simCos) <- paste(species)
  ## count pass
  Pass <- Pass + 1
  ## calculate Jaccard coefficients
  N <- intensityMatrix(peaks)
  rownames(N) <- paste(species)
  N[N>0] <- 1
  N[is.na(N)] <- 0
```

```
  k <- distance((N), method = "jaccard")
  Rich <- ncol(N)
  richE <- sum(N[1,])
  # calculate BTS match
# create Jaccard similarity
rownames(k) <- paste(species)
colnames(k) <- paste(species)
Cor12 <- 0
k2 <- 1 - k
# create dataframe of simCos pairwise comparisons
  xySC <- t(combn(colnames(simCos), 2))
  xyS <- data.frame(xySC, dist=simCos[xySC])
  names(xyS)[3] <- "distS"
  xyS$sPair <- paste(xyS$X1,xyS$X2,sep="-")
#  head(xyS)
  xyJC <- t(combn(colnames(k2), 2))
  xyJ <- data.frame(xyJC, distJ=k2[xyJC])
#  head(xyJ)
  ## merge jaccard and simCos
  xyJS <- data.frame(xyS,xyJ[3])
# calculate Yo
#y0 <- xyJS[ which(xyJS$distJ < 0.01),]
# hist(y0$distS)
#y0 <- exp(mean(log(y0$distS)))
y0 <- 0
Km <- 0.2
# plot predicted simCos
xyJS$cosPred <- y0 + (xyJS$distJ/(Km+xyJS$distJ))
# plot.default(xyJS$distJ, xyJS$distS, xlab = "Jaccard", ylab = "SimCos")
# plot.default(xyJS$distS, xyJS$cosPred, xlab = "Jaccard", ylab = "SimCos")
# predict matrix
kW <- y0 + (k2/(Km+k2))
# weigh matrix
cosW <- (simCos + kW)/2
# create dataframe of weighted pairwise comparisons
  xyW <- t(combn(colnames(cosW), 2))
  xyW <- data.frame(xyW, distCW=cosW[xySC])
  names(xyW)[3] <- "distWy"
xyS <- data.frame(xyJS, xyW[3])
# merge reference and simCos
mRM <- merge(xyRef, xyS, by, by.x="sPair", by.y="sPair", sort = TRUE)
# calculate mass spec similarity within putative MTUs
mRMref <- mRM[ which(mRM$distWref > 0.7),]
distJ <- mean(mRMref$distJ.y)
fit16W <- min(mRMref$distWy)
distS <- mean(mRMref$distS.y)
distW <- mean(mRMref$distWy)
# calculate mass spec similarity between phyla
mRMp <- mRM[ which(mRM$distWref < 0.4),]
outJ <- mean(mRMp$distS.y)
outW <- mean(mRMp$distWy)
fitTry <- distW - outW
  ## name each data value
  SNRalgn <- c(hwSmth, rmvbI, hwA, snrA, tolA, hwD, snrD, Rich, Cor12, richE, K12, Pass, M12, outJ, fitTry,
fit16W, outJ, Km, y0, distJ, distS, distW)
  names(SNRalgn) <- c("hwSmth", "rmvbI", "hwAlgn", "snrAlgn", "tolAlgn", "hwDtct", "snrDtct", "peaks", "Cor
```

```
12", "RichE", "K12", "Pass", "MaxRep", "out16S", "fitTry", "fit16W", "out16W", "Km", "y0", "distJ", "dist
S", "distW")
  B = matrix(SNRalgn, nrow=22, ncol=1)
  Dq <- cbind(B, Dq)
  M12 <- max(Dq[22,])
#saveRDS(Dq, "DqJSCwgs3.Rds")
    # set lower pass number for markdown
  if (Pass > 9) {break}
}# end of align loop
```

```
## Error in FUN(X[[i]], ...) :
##   Could not match enough peaks in spectrum42to the reference peaks.
## 2 matches required, just 1 found.
## Error in FUN(X[[i]], ...) :
##   Could not match enough peaks in spectrum35to the reference peaks.
## 2 matches required, just 1 found.
## Error in FUN(X[[i]], ...) :
##   Could not match enough peaks in spectrum35to the reference peaks.
## 2 matches required, just 1 found.
## Error in FUN(X[[i]], ...) :
##   Could not match enough peaks in spectrum34to the reference peaks.
## 2 matches required, just 1 found.
## Error in FUN(X[[i]], ...) :
##   Could not match enough peaks in spectrum35to the reference peaks.
## 2 matches required, just 1 found.
## Error in FUN(X[[i]], ...) :
##   Could not match enough peaks in spectrum2to the reference peaks.
## 2 matches required, just 1 found.
```

```
write.csv(as.matrix(k), "k.csv")
#write.csv(as.matrix(t(Dq)), "Dq.csv")
DFq <- as.data.frame(t(Dq))
plot.default(DFq$Pass, DFq$MaxRep, xlab = "Try", ylab = "distW")
```

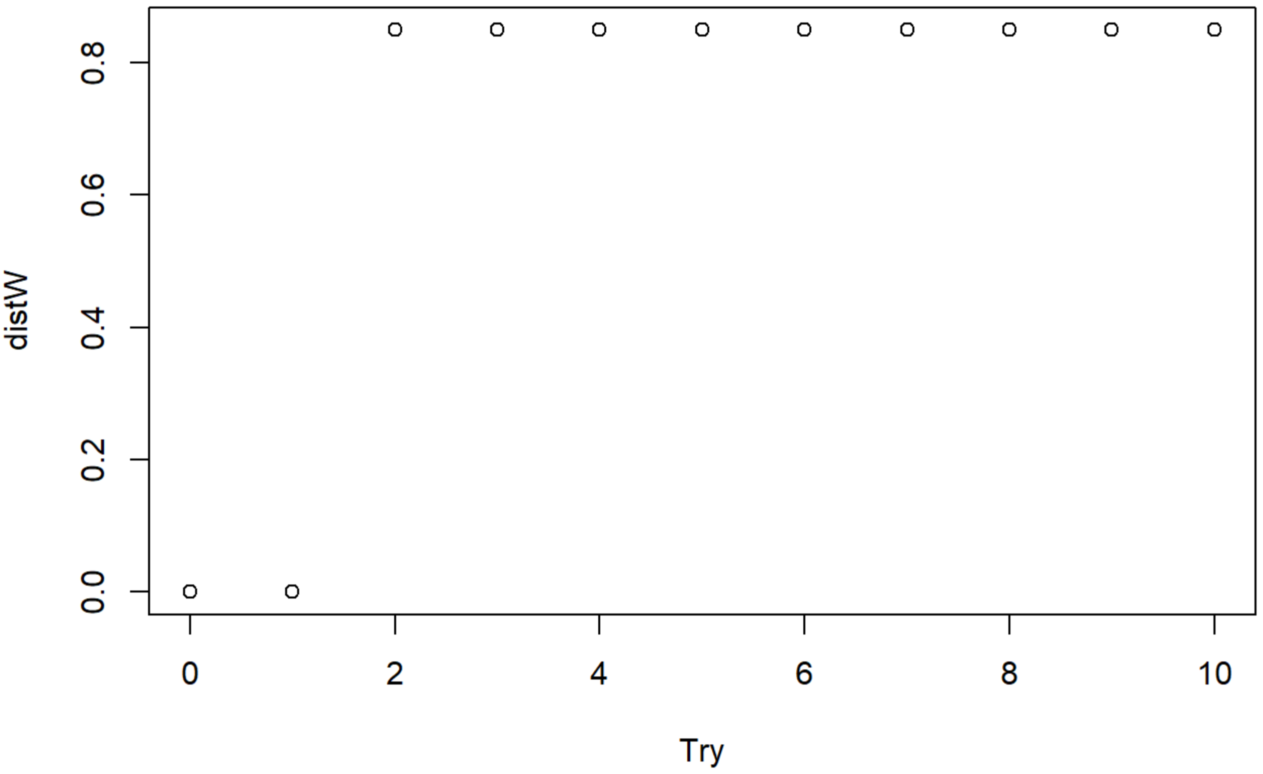

Import alignment parameters from larger run and plot jaccard within BTS versus peaks

```
DFq <- readRDS("DqJSCwgs2.Rds")
DFq <- as.data.frame(t(DFq))
DFq <- DFq[ which(DFq$K12 > 0.1),]
DFq <- DFq[ which(DFq$distJ > 0.01),]
plot.default(DFq$peaks, DFq$distJ, xlab = "Peaks", ylab = "Shared Peaks")
```

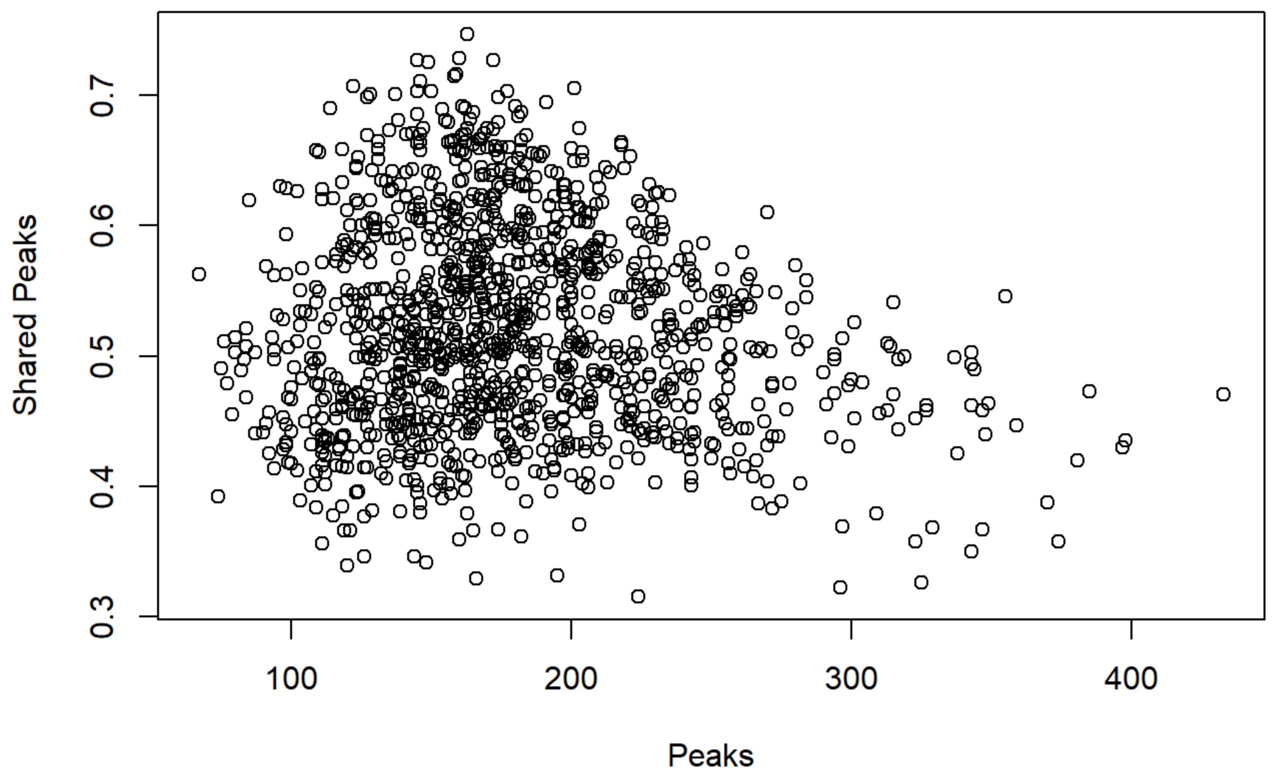

select best fit WW

```
DFj <- DFq[ which(DFq$distW > 0.01),]
DFj <- DFj[ which(DFj$peaks > 120),]
plot.default(DFj$distW, DFj$fitTry, xlab = "Similarity within Genera", ylab = "Difference between Genera")
```

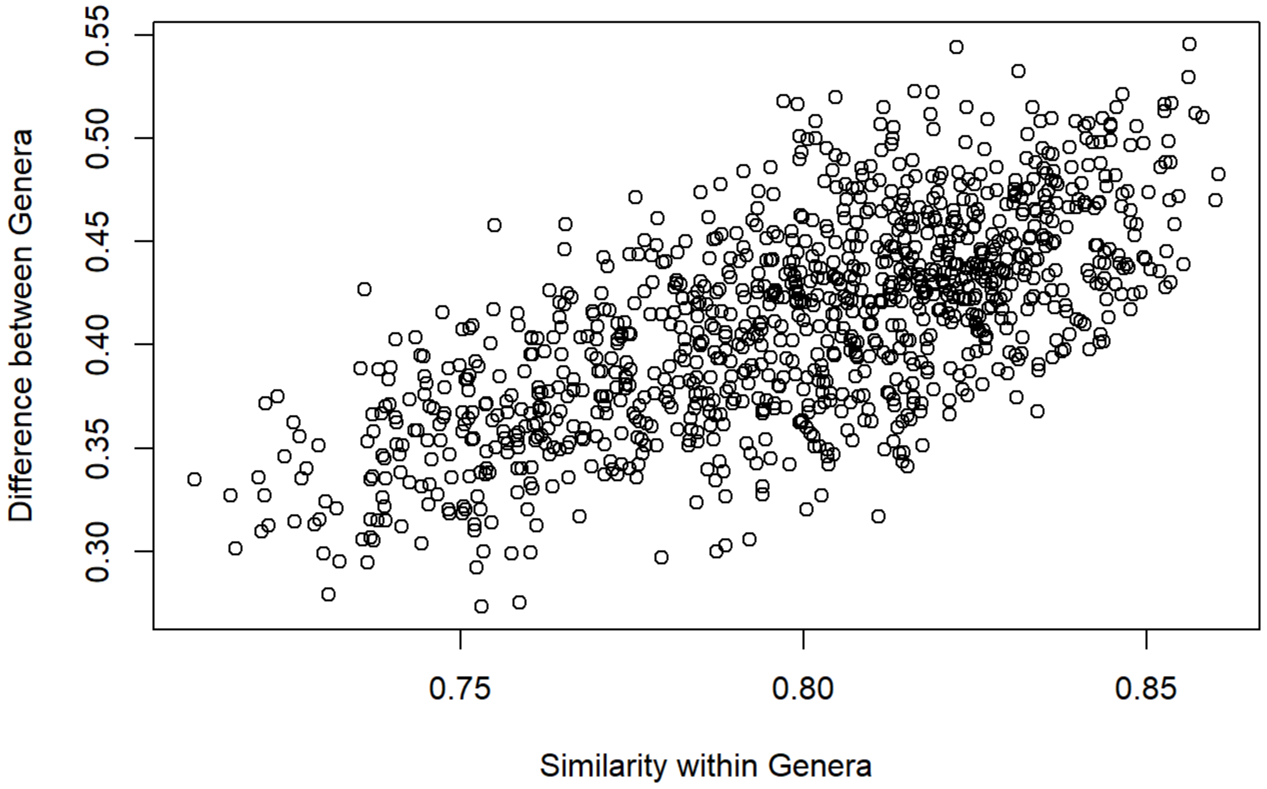

select best fit

```
plot.default(DFj$peaks, DFj$distJ, xlab = "Peaks", ylab = "Shared peaks")
```

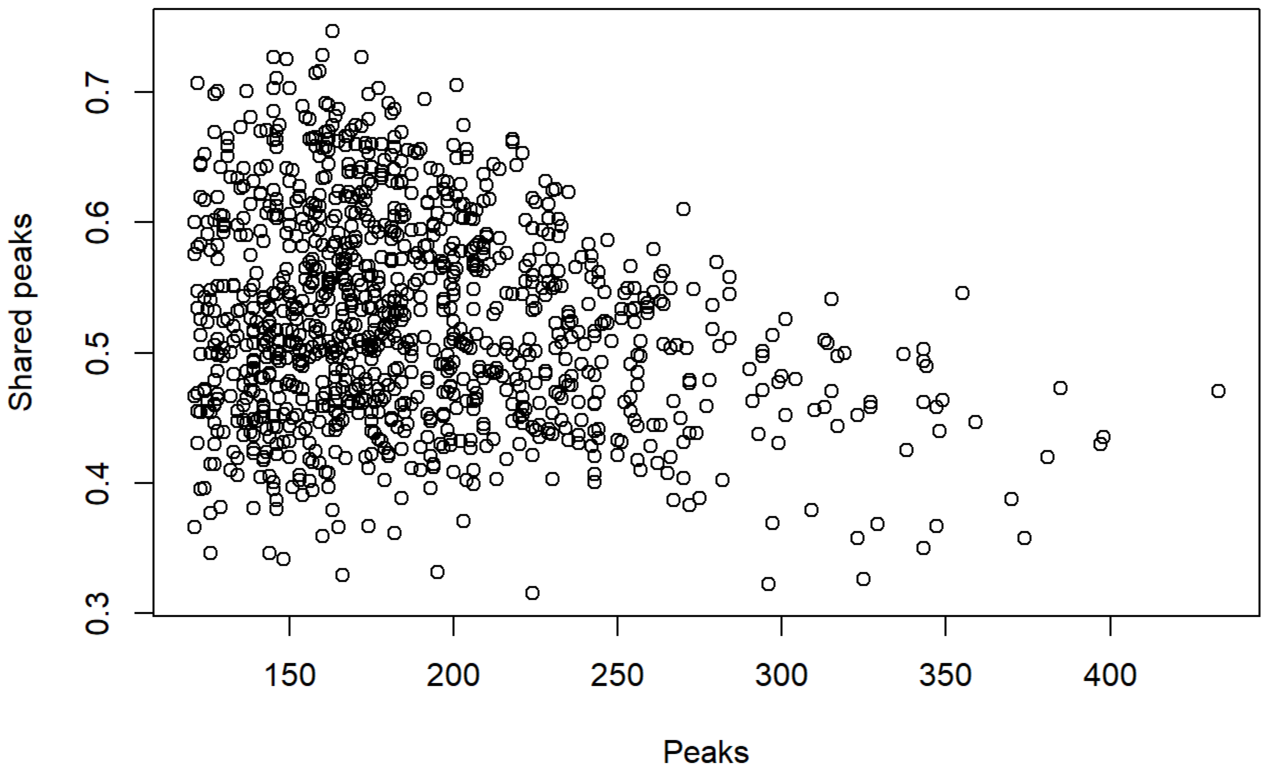

select best fit

```
DF <- DFj[order(-DFj$distW, -DFj$peaks),]
dim(DF)
```

```
## [1] 1066   22
```

```
hwSmth <- DF$hwSmth[1]
rmvbI <- DF$rmvbI[1]
hwA <-  DF$hwA[1]
snrA <-  DF$snrA[1]
tolA <- DF$tolA[1]
hwD <- DF$hwD[1]
snrD <- DF$snrD[1]
hwSmth
```

```
## [1] 60
```

```
rmvbI
```

```
## [1] 75
```

```
hwA
```

```
## [1] 90
```

```
snrA
```

```
## [1] 2.4
```

```
tolA
```

```
## [1] 0.027
```

```
hwD
```

```
## [1] 100
```

```
snrD
```

```
## [1] 6
```

align spectra with optimized parameters

```
spectra <- smoothIntensity(spectraJ, method="SavitzkyGolay", halfWindowSize=hwSmth)
spectra <- removeBaseline(spectra, method="SNIP",iterations=rmvbI)
spectraT <- calibrateIntensity(spectra, method="TIC")
spectra <- try(alignSpectra
               (spectraT, halfWindowSize=hwA, SNR=snrA, reference = rR,
                 tolerance=tolA, warpingMethod="lowess"), silent = FALSE)
if ('try-error' %in% class(spectra)) next
##---metadata
LB  <- length(spectra)
metaData(spectra[[LB]])$sampleName
```

```
## [1] "d2069_SDA_1"
```

```
spots <- sapply(spectra, function(x)metaData(x)$spot)
# species <- sapply(spectra, function(x)metaData(x)$sampleName)
# name all spectra by isolate (w/o regard to when it was run)
species <- speciesNamesJ$aaiCode
avgSpectra <-
  averageMassSpectra((spectra), labels=paste0(species))
## detectPeaks
try(peaks <- detectPeaks(avgSpectra, SNR=snrD, halfWindowSize=hwD), silent = FALSE)
if ('try-error' %in% class(avgSpectra)) next
##--export peaks
peaks <- binPeaks(peaks)
# save peaks
spots <- sapply(avgSpectra, function(x)metaData(x)$spot)
species <- labels(spots)
species <- factor(species)
n <- length(avgSpectra)
featureMatrix <- intensityMatrix(peaks, avgSpectra)
rownames(featureMatrix) <- paste(species)
peaksNew <- intensityMatrix(peaks)
rownames(peaksNew) <- paste(species)
##--write matrix
FMtrnsp <- t(featureMatrix)
#write.csv(FMtrnsp, "FMtJSC.csv")
nrow(FMtrnsp)
```

```
## [1] 160
```

```
# calcualte cos simmilarity
simCos <- coop::cosine(FMtrnsp)
#rownames(simCos) <- paste(species)
## calculate Jaccard coefficients
N <- intensityMatrix(peaks)
#rownames(N) <- paste(species)
N[N>0] <- 1
N[is.na(N)] <- 0
k <- distance((N), method = "jaccard")
```

```
## Metric: 'jaccard'; comparing: 15 vectors.
```

```
#rownames(k) <- paste(species)
Rich <- ncol(N)
richE <- sum(N[1,])
#n < nrow(N)
pvJ <- pvclust(FMtrnsp,
              method.hclust="ward.D2",
              method.dist="euclidean", nboot=1000)
```

```
## Bootstrap (r = 0.5)... Done.
## Bootstrap (r = 0.6)... Done.
## Bootstrap (r = 0.7)... Done.
## Bootstrap (r = 0.8)... Done.
## Bootstrap (r = 0.9)... Done.
## Bootstrap (r = 1.0)... Done.
## Bootstrap (r = 1.1)... Done.
## Bootstrap (r = 1.2)... Done.
## Bootstrap (r = 1.3)... Done.
## Bootstrap (r = 1.4)... Done.
```

```
saveRDS(pvJ, "jscPVJ.rds")
plot(pvJ, print.num=TRUE)
pvrect(pvJ, alpha=0.99)
```

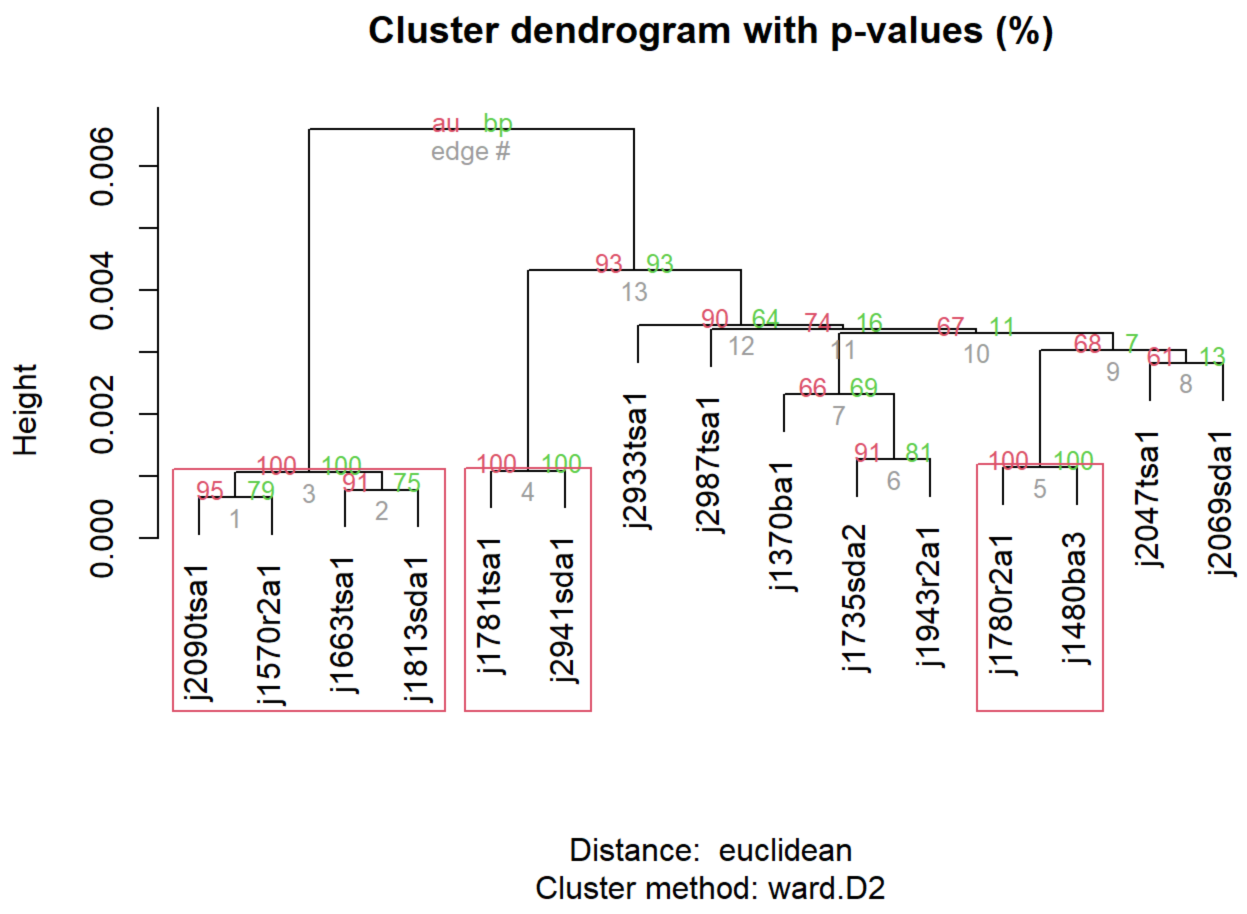

archive plot

```
svg(file = "aniJSC_4.svg", width = 11, height = 8.5)
plot(pvJ, print.num=TRUE)
pvrect(pvJ, alpha = 0.99)
dev.off()
```

```
## png
##   2
```

create dataframe

```
xySC <- t(combn(colnames(simCos), 2))
xyS <- data.frame(xySC, dist=simCos[xySC])
names(xyS)[3] <- "distS"
xyS$sPair <- paste(xyS$X1,xyS$X2,sep="-")
head(xyS)
```

```
##         X1        X2     distS              sPair
## 1 j1370ba1 j1663tsa1 0.3714967 j1370ba1-j1663tsa1
## 2 j1370ba1 j1735sda2 0.7615561 j1370ba1-j1735sda2
## 3 j1370ba1 j1780r2a1 0.5389529 j1370ba1-j1780r2a1
## 4 j1370ba1 j1781tsa1 0.4405024 j1370ba1-j1781tsa1
## 5 j1370ba1 j1813sda1 0.4073187 j1370ba1-j1813sda1
## 6 j1370ba1 j1943r2a1 0.7045342 j1370ba1-j1943r2a1
```

```
#
Km <- 0.2
y0 <- 0
k2 <- 1 - k
kW <- y0 + (k2/(Km+k2))
# weigh matrix
cosW <- (simCos + kW)/2
# create dataframe of weighted pairwise comparisons
xyW <- t(combn(colnames(cosW), 2))
xyW <- data.frame(xyW, distCW=cosW[xySC])
names(xyW)[3] <- "distW"
xyS <- data.frame(xyS, xyW[3])
head(xyS)
```

```
##         X1        X2     distS              sPair     distW
## 1 j1370ba1 j1663tsa1 0.3714967 j1370ba1-j1663tsa1 0.3780561
## 2 j1370ba1 j1735sda2 0.7615561 j1370ba1-j1735sda2 0.6897668
## 3 j1370ba1 j1780r2a1 0.5389529 j1370ba1-j1780r2a1 0.4778098
## 4 j1370ba1 j1781tsa1 0.4405024 j1370ba1-j1781tsa1 0.4077512
## 5 j1370ba1 j1813sda1 0.4073187 j1370ba1-j1813sda1 0.3801300
## 6 j1370ba1 j1943r2a1 0.7045342 j1370ba1-j1943r2a1 0.6678011
```

add dataframe of jaccards

```
xyJC <- t(combn(colnames(k2), 2))
xyJ <- data.frame(xyJC, distJ=k2[xyJC])
head(xyJ)
```

```
##   X1 X2     distJ
## 1 v1 v2 0.1250000
## 2 v1 v3 0.3235294
## 3 v1 v4 0.1428571
## 4 v1 v5 0.1200000
## 5 v1 v6 0.1090909
## 6 v1 v7 0.3421053
```

```
## merge jaccard and simCos
xyJS <- data.frame(xyS,xyJ[3])
head(xyJS)
```

```
##         X1        X2     distS              sPair     distW     distJ
## 1 j1370ba1 j1663tsa1 0.3714967 j1370ba1-j1663tsa1 0.3780561 0.1250000
## 2 j1370ba1 j1735sda2 0.7615561 j1370ba1-j1735sda2 0.6897668 0.3235294
## 3 j1370ba1 j1780r2a1 0.5389529 j1370ba1-j1780r2a1 0.4778098 0.1428571
## 4 j1370ba1 j1781tsa1 0.4405024 j1370ba1-j1781tsa1 0.4077512 0.1200000
## 5 j1370ba1 j1813sda1 0.4073187 j1370ba1-j1813sda1 0.3801300 0.1090909
## 6 j1370ba1 j1943r2a1 0.7045342 j1370ba1-j1943r2a1 0.6678011 0.3421053
```

create reference

```
hist(xyJS$distW)
```

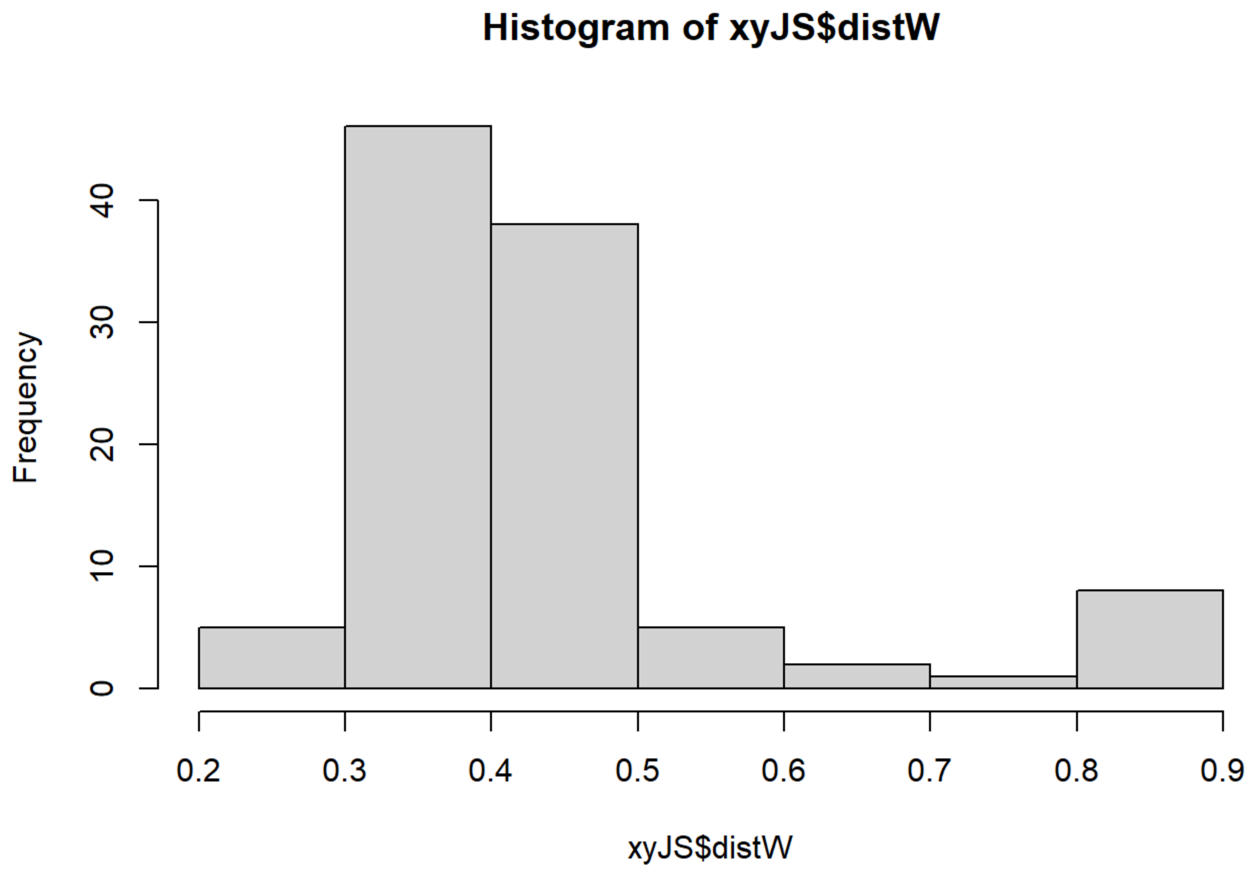

plot weighted

```
#write.csv(xyJS, "xyJSC.csv")
plot.default(xyJS$distJ, xyJS$distW, xlab = "Jaccard", ylab = "SimCosW")
```

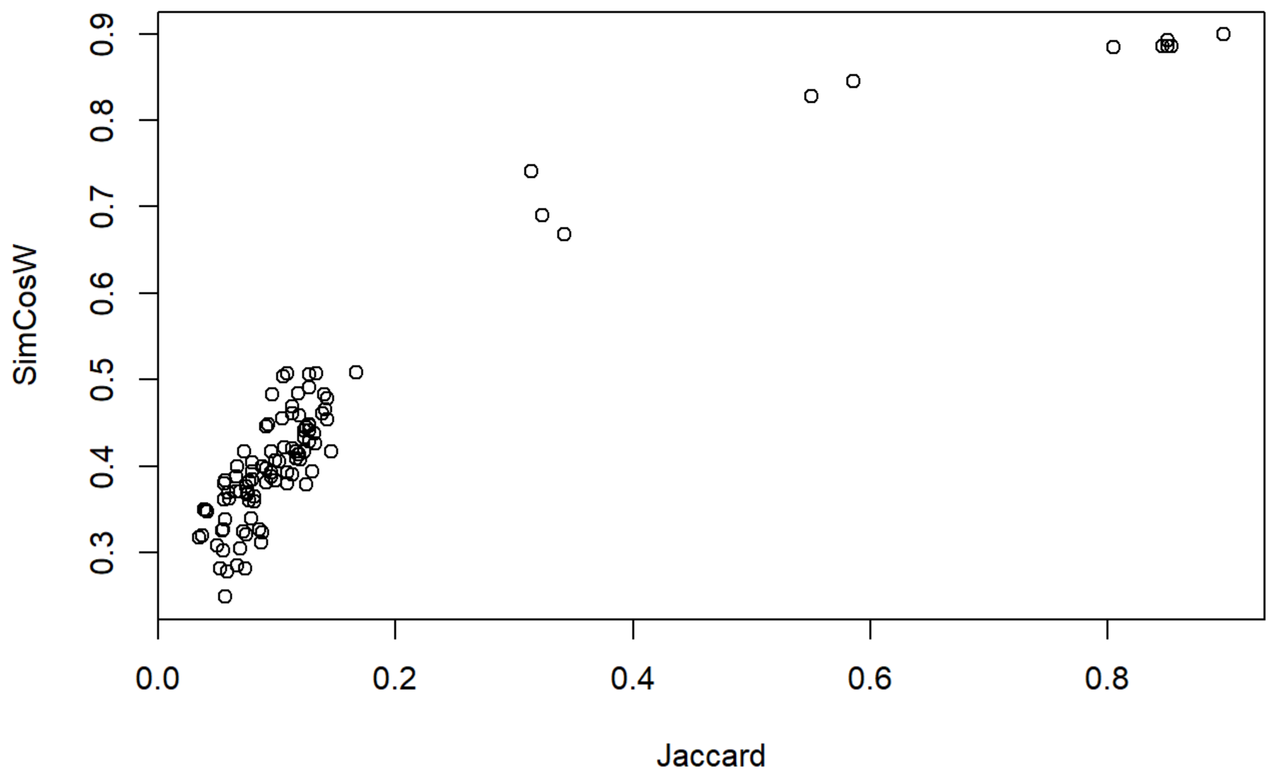

merge

simCosW and AAI.

```
AAident <- read.table("aaiJSC.csv", sep=",", header = TRUE, check.names = TRUE)
#xy <- t(combn(colnames(AAident), 2))
#xyA <- data.frame(xy, dist=AAident[xy])
AAident$AApair <- paste(AAident$Label.1,AAident$Label.2,sep="-")
#names(xyR)[3] <- "distR"
tail(AAident)
```

```
##       Label.1   Label.2       AAI CDS.count.1 CDS.count.2 Matched.count
## 220 j2987tsa1 j2047tsa1  60.17229        3868        3769          1422
## 221 j2987tsa1 j2069sda1  63.02733        3868        5613          2089
## 222 j2987tsa1 j2090tsa1  62.48711        3868        5809          1912
## 223 j2987tsa1 j2933tsa1  63.95355        3868        5480          1973
## 224 j2987tsa1 j2941sda1  57.01626        3868        6051          1467
## 225 j2987tsa1 j2987tsa1 100.00000        3868        3868          3840
##     Proteome.cov. ID.param. Cov..param.              AApair
## 220      0.372398       0.4         0.5 j2987tsa1-j2047tsa1
## 221      0.440671       0.4         0.5 j2987tsa1-j2069sda1
## 222      0.395164       0.4         0.5 j2987tsa1-j2090tsa1
## 223      0.422122       0.4         0.5 j2987tsa1-j2933tsa1
## 224      0.295796       0.4         0.5 j2987tsa1-j2941sda1
## 225      0.992761       0.4         0.5 j2987tsa1-j2987tsa1
```

merge simCosW and AAI.

```
mRM <- merge(AAident, xyJS, by, by.x="AApair", by.y="sPair", sort = TRUE)
head(mRM)
```

```
##             AApair  Label.1   Label.2      AAI CDS.count.1 CDS.count.2
## 1  j1370ba1-j1480ba3 j1370ba1  j1480ba3 63.92091        3685        4114
## 2 j1370ba1-j1570r2a1 j1370ba1 j1570r2a1 70.35712        3685       10618
## 3 j1370ba1-j1663tsa1 j1370ba1 j1663tsa1 61.97385        3685        5692
## 4 j1370ba1-j1735sda2 j1370ba1 j1735sda2 94.48868        3685        4176
## 5 j1370ba1-j1780r2a1 j1370ba1 j1780r2a1 63.63808        3685        4110
## 6 j1370ba1-j1781tsa1 j1370ba1 j1781tsa1 56.85446        3685        6338
##   Matched.count Proteome.cov. ID.param. Cov..param.       X1        X2
## 1          1846      0.473394       0.4         0.5 j1370ba1  j1480ba3
## 2          2808      0.392645       0.4         0.5 j1370ba1 j1570r2a1
## 3          1864      0.397569       0.4         0.5 j1370ba1 j1663tsa1
## 4          3380      0.859941       0.4         0.5 j1370ba1 j1735sda2
## 5          1925      0.493906       0.4         0.5 j1370ba1 j1780r2a1
## 6          1481      0.295520       0.4         0.5 j1370ba1 j1781tsa1
##       distS     distW     distJ
## 1 0.5614879 0.5080167 0.1666667
## 2 0.4128953 0.4169740 0.1454545
## 3 0.3714967 0.3780561 0.1250000
## 4 0.7615561 0.6897668 0.3235294
## 5 0.5389529 0.4778098 0.1428571
## 6 0.4405024 0.4077512 0.1200000
```

```
#write.csv(as.matrix(mRM), "mRMjsc.csv")
plot.default(mRM$distJ, mRM$AAI, xlab = "distJ", ylab = "Amino acid identity")
```

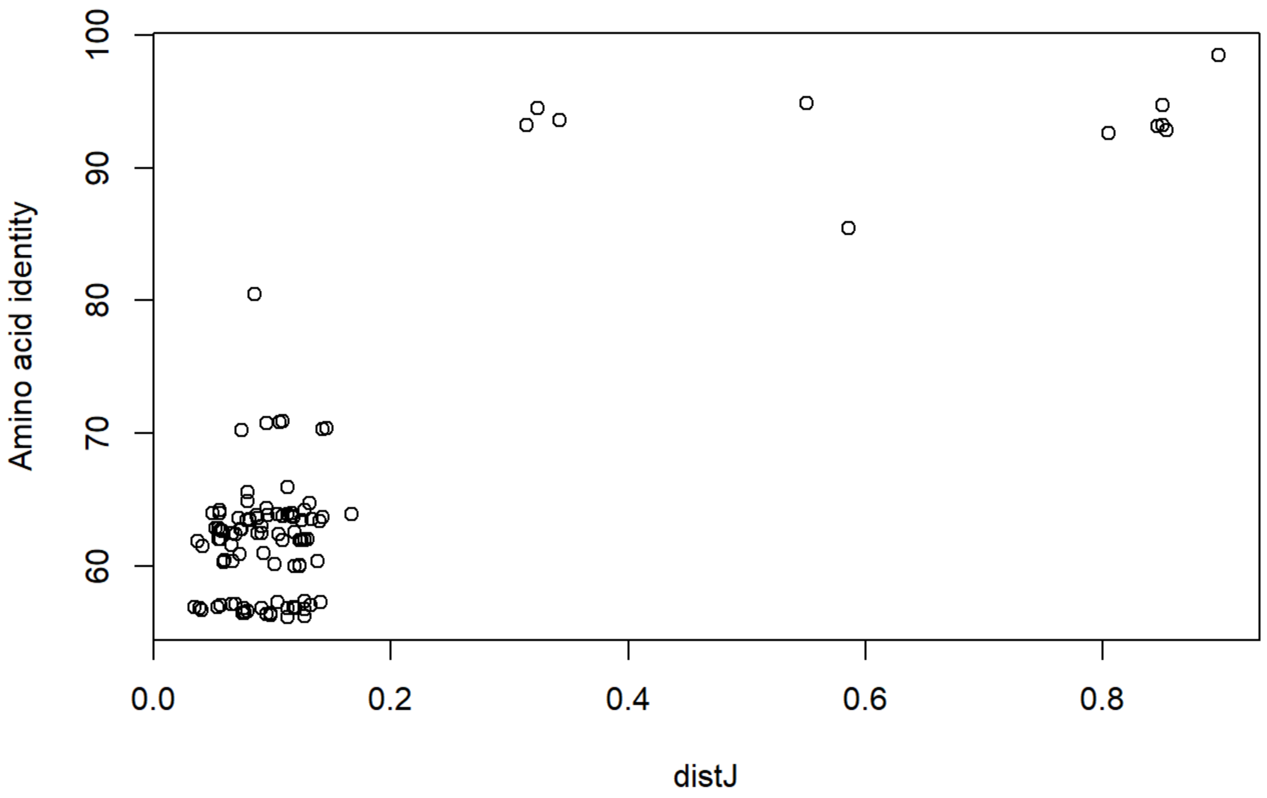

plot sim cos.

```
plot.default( mRM$AAI, mRM$distW, xlab = "Amino acid similarity", ylab = "MALDI-TOF similarity")
```

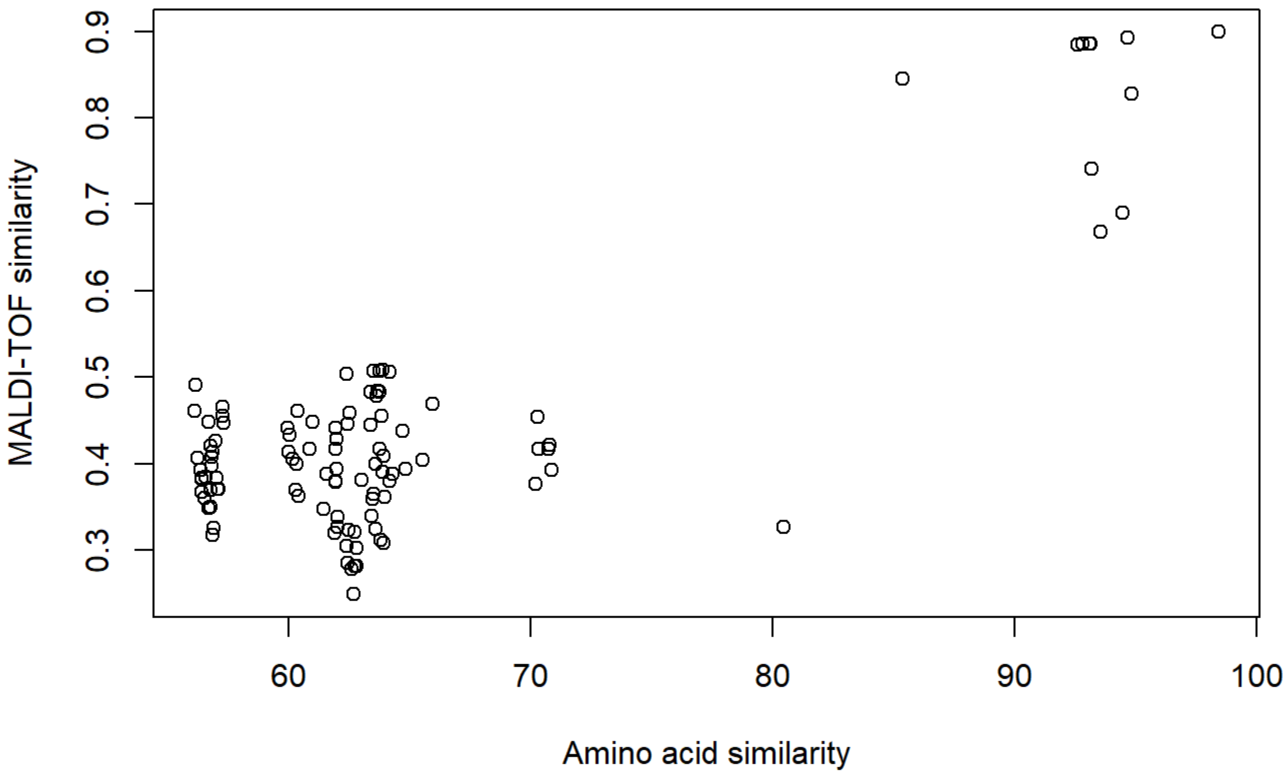

cluster isolates into MTUs based on 0.65 similarity

```
disCos <- 1-cosW
hc <- hclust(as.dist(disCos), method="ward.D2")
mycut <- cutree(hc, h=0.20)
xCut <- as.matrix(mycut)
colnames(xCut) <- paste("MTU")
xCut <- as.matrix(mycut)
colnames(xCut) <- paste("MTU")
head(xCut)
```

```
##           MTU
## j1370ba1    1
## j1663tsa1   2
## j1735sda2   3
## j1780r2a1   4
## j1781tsa1   5
## j1813sda1   2
```

calculate SNR for avgspectra

```
spots <- sapply(avgSpectra, function(x)metaData(x)$spot)
#species <- sapply(avgSpectra, function(x)metaData(x)$sampleName)
species <- labels(spots)
species <- factor(species)
# reset matrix
x <- c(0.1:0.6)
E <- matrix(x, nrow=6, ncol=1)
rownames(E) <- c("speciesE", "tryE", "noiseE", "signal", "SNR", "peaks")
## reset counter
tryE <- c(0)
n <- length(avgSpectra)
n
```

```
## [1] 15
```

run SNR loop on avgSpectra

```
repeat{
  tryE <- tryE + 1
  noise <- estimateNoise(avgSpectra[[tryE]])
 # plot(avgSpectra[[tryE]], xlim=c(2000, 12000), ylim=c(0, 0.002))
 # points(peaks[[tryE]], col="red", pch=4)
##--generate matrix
featureMatrix <- intensityMatrix(peaks, avgSpectra)
  rownames(featureMatrix) <- paste(species, spots)
  nrow(featureMatrix)
  ## calculate Signal
  noise <- estimateNoise(avgSpectra[[tryE]])
  head(noise)
speciesE <- rownames(featureMatrix)
speciesE <- speciesE[tryE]
  EaPeaks <- intensityMatrix(peaks[tryE])
  richE <- ncol(EaPeaks)
  sorted <- EaPeaks[order(EaPeaks, decreasing = TRUE)]
  signal <- median((sorted[1:10]))
  noiseE <- noise[1, 2]
  SNRe <- (signal/noiseE)
  ## name each data value
  SNRall <- c(speciesE, tryE, noiseE, signal, SNRe, richE)
  names(SNRall) <- c("speciesE", "tryE", "noiseE", "signal", "SNR", "peaks")
  C = matrix(SNRall, nrow=6, ncol=1)
  E <- cbind(C, E)
 # plot.default(E[1,], E[2,], xlab = "Try", ylab = "Duplicate")
  if (tryE > (n-1)) {break}}
 plot(avgSpectra[[tryE]], xlim=c(2000, 12000), ylim=c(0, 0.002))
 points(peaks[[tryE]], col="red", pch=4)
```

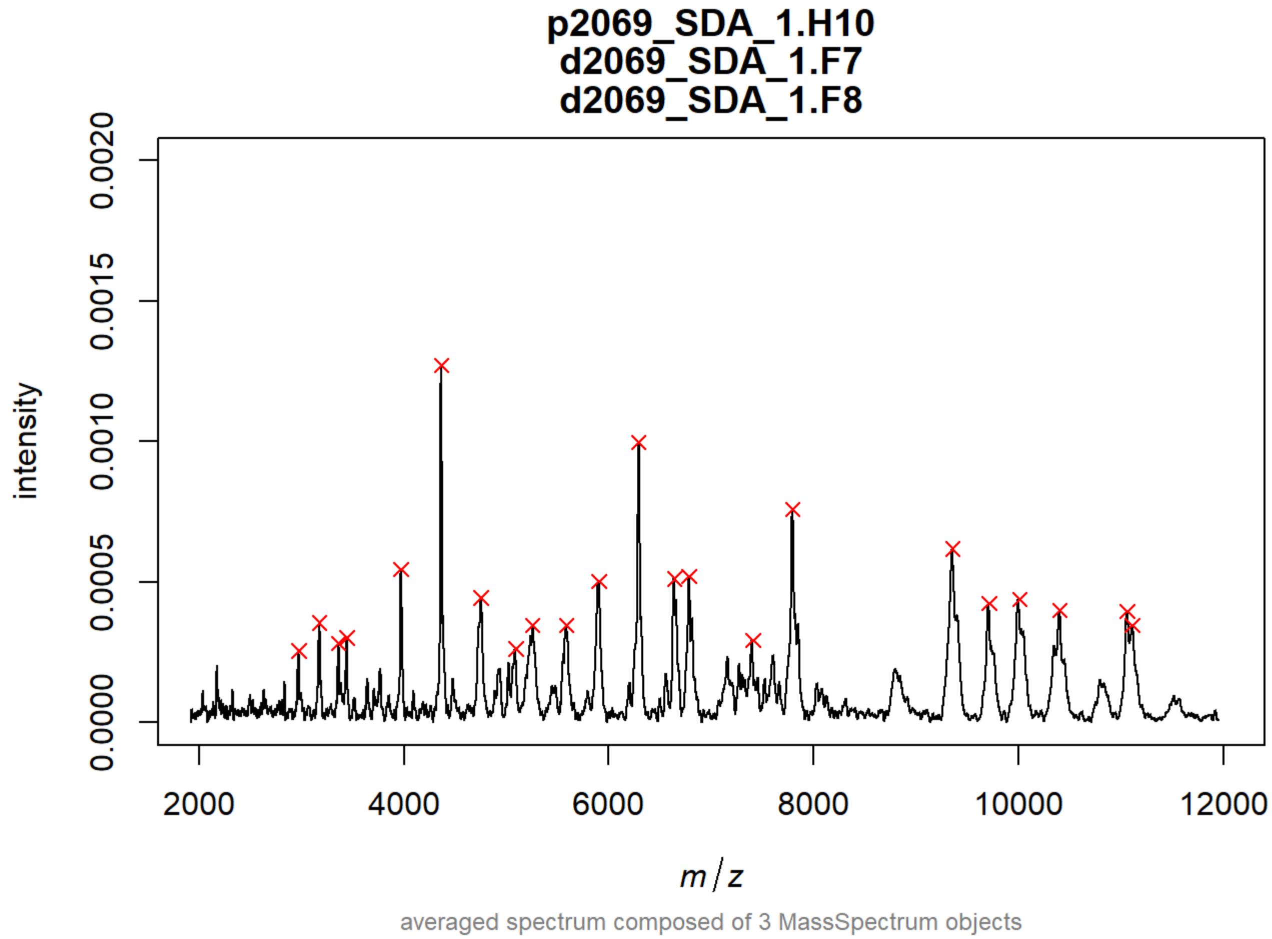

sort SNR

```
F <- t(E[,1:n])
F[is.na(F)] = 0
F <- as.data.frame(F)
F$tryE <- as.numeric(F$tryE)
F$noiseE <- as.numeric(F$noiseE)
F$signal<- as.numeric(F$signal)
F$SNR <- as.numeric(F$SNR)
F$peaks <- as.numeric(F$peaks)
F <- F[order(F$tryE),]
row.names(F) <- F$tryE
head(F)
```

```
##                                                                         speciesE
## 1  j1370ba1 c("D1", "D2", "D3", "A5", "A6", "A7", "A8", "A9", "H6", "E11", "E12")
## 2             j1663tsa1 c("D11", "D12", "D9", "C1", "C2", "C4", "C5", "C5", "C6")
## 3    j1735sda2 c("A10", "A11", "A12", "C11", "C12", "D1", "D2", "H9", "F5", "F6")
## 4          j1780r2a1 c("B5", "B7", "B8", "D4", "D5", "D6", "D7", "H7", "F1", "F2")
## 5 j1781tsa1 c("B1", "B2", "B3", "D11", "D12", "D9", "E1", "H3", "E4", "E5", "E6")
## 6       j1813sda1 c("H1", "H2", "H4", "E3", "E4", "E5", "E6", "C10", "C11", "C9")
##   tryE       noiseE       signal      SNR peaks
## 1    1 3.624349e-05 0.0005654195 15.60058    25
## 2    2 3.278335e-05 0.0007225351 22.03970    38
## 3    3 4.126824e-05 0.0004874618 11.81203    20
## 4    4 3.454488e-05 0.0006048813 17.51001    31
## 5    5 3.302961e-05 0.0007569329 22.91680    31
## 6    6 3.668716e-05 0.0006410124 17.47239    36
```

```
write.csv(F, "FjscMJ.csv")
```

plot avg SNR

```
plot.default(F$SNR, F$peaks, xlab = "SNR", ylab = "peaks")
```

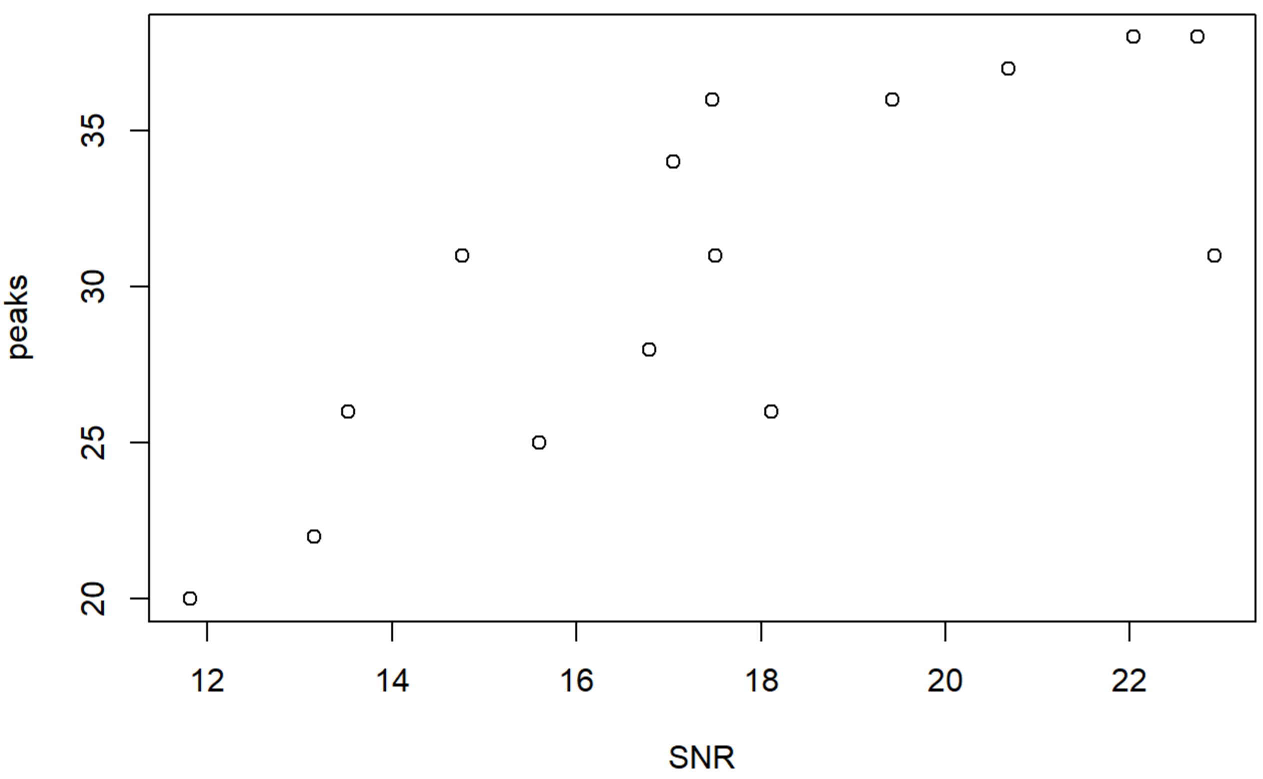

merge SNR and MTUs

```
xCut <- as.matrix(mycut)
colnames(xCut) <- paste("MTU")
# merge SNR and MTU
FM <- as.matrix(F[,2:6])
row.names(FM) <- row.names(xCut)
FM <- cbind(FM, xCut)
dim(FM)
```

```
## [1] 15  6
```

```
#
FMsort <- FM[ order(row.names(FM)), ]
write.csv(as.matrix(FMsort), "FMsortJSCmjd.csv")
getwd()
```

```
## [1] "C:/Users/mglam/Dropbox/BIOINFO/Rscripts/ANIjsc"
```

```
head(FMsort)
```

```
##           tryE       noiseE       signal      SNR peaks MTU
## j1370ba1     1 3.624349e-05 0.0005654195 15.60058    25   1
## j1480ba3    13 3.635401e-05 0.0005368522 14.76734    31   4
## j1570r2a1   14 3.111311e-05 0.0007072997 22.73317    38   2
## j1663tsa1    2 3.278335e-05 0.0007225351 22.03970    38   2
## j1735sda2    3 4.126824e-05 0.0004874618 11.81203    20   3
## j1780r2a1    4 3.454488e-05 0.0006048813 17.51001    31   4
```

import Bruker scores and IDs

```
brukerID <- read.table("BrukerJSCall.csv", sep=",", header = TRUE, check.names = TRUE, row.names = 1)
SNRbruker <- cbind(FMsort, brukerID)
# add number of reps for each species
fact = interaction(SNRbruker[, (c("MTU"))])
SNRbruker$count = table(fact)[fact]
head(SNRbruker)
```

```
##           tryE       noiseE       signal      SNR peaks MTU          BrukerID
## j1370ba1     1 3.624349e-05 0.0005654195 15.60058    25   1 Bacillus safensis
## j1480ba3    13 3.635401e-05 0.0005368522 14.76734    31   4   Bacillus flexus
## j1570r2a1   14 3.111311e-05 0.0007072997 22.73317    38   2   Bacillus cereus
## j1663tsa1    2 3.278335e-05 0.0007225351 22.03970    38   2   Bacillus cereus
## j1735sda2    3 4.126824e-05 0.0004874618 11.81203    20   3  Bacillus pumilus
## j1780r2a1    4 3.454488e-05 0.0006048813 17.51001    31   4   Bacillus flexus
##           BrukerScore count
## j1370ba1        2.030     1
## j1480ba3        2.380     2
## j1570r2a1       2.279     4
## j1663tsa1       2.310     4
## j1735sda2       2.060     1
## j1780r2a1       2.320     2
```

```
#write.csv(as.matrix(SNRbruker), "snrBrukerJSC.csv")
```

plot SNR vs BrukerScore

```
plot.default(SNRbruker$SNR, SNRbruker$BrukerScore, xlab = "SNR", ylab = "BrukerScore")
```

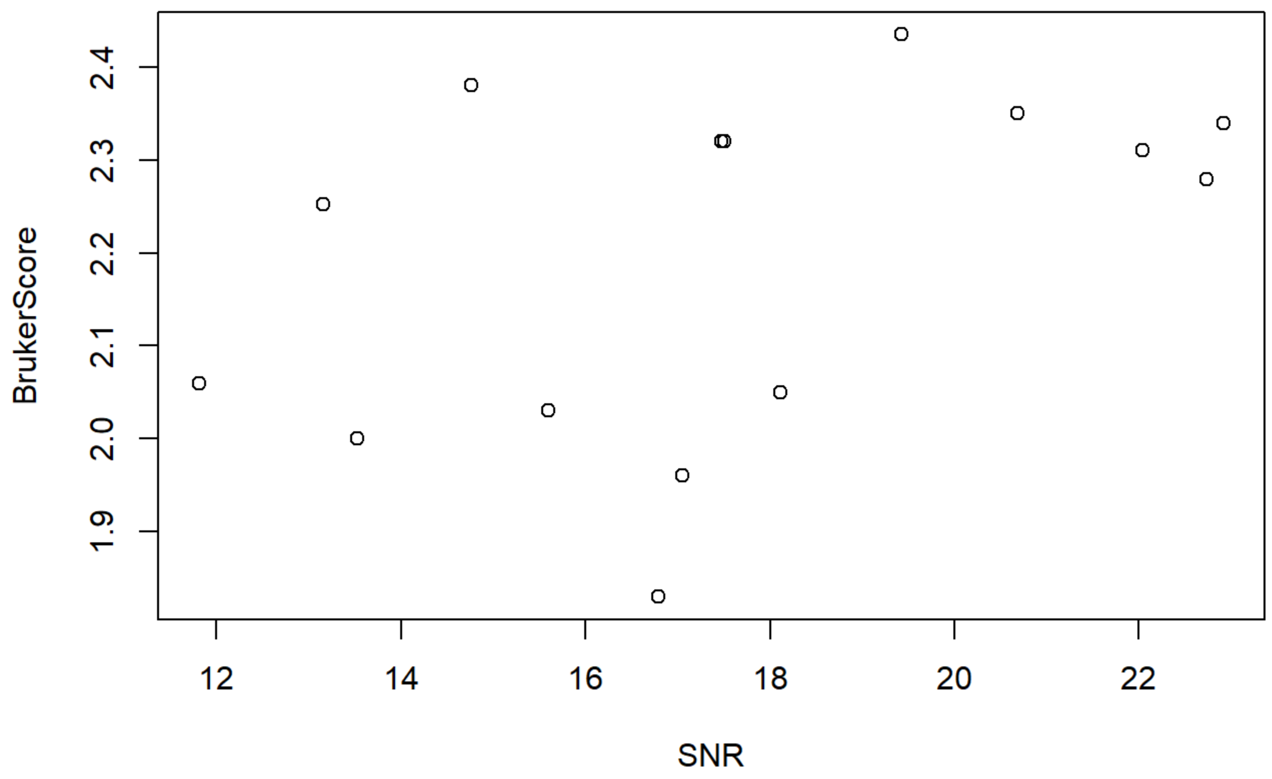

table the number of MTUs

```
mtu <- with(SNRbruker, table(MTU))
mtu <- as.data.frame(mtu[-c(1)])
mtu <- mtu$Freq
length(mtu)
```

```
## [1] 9
```

```
head(mtu)
```

```
## [1] 4 1 2 2 1 1
```

Run rarefaction analysis

```
out <- iNEXT(mtu, q=0, datatype="abundance")
# plot collectors curve
m <- ggiNEXT(out, type=1)
```

```
## Warning in ggiNEXT.iNEXT(out, type = 1): invalid color.var setting, the iNEXT
## object do not consist multiple assemblages, change setting as Order.q
```

```
m
```

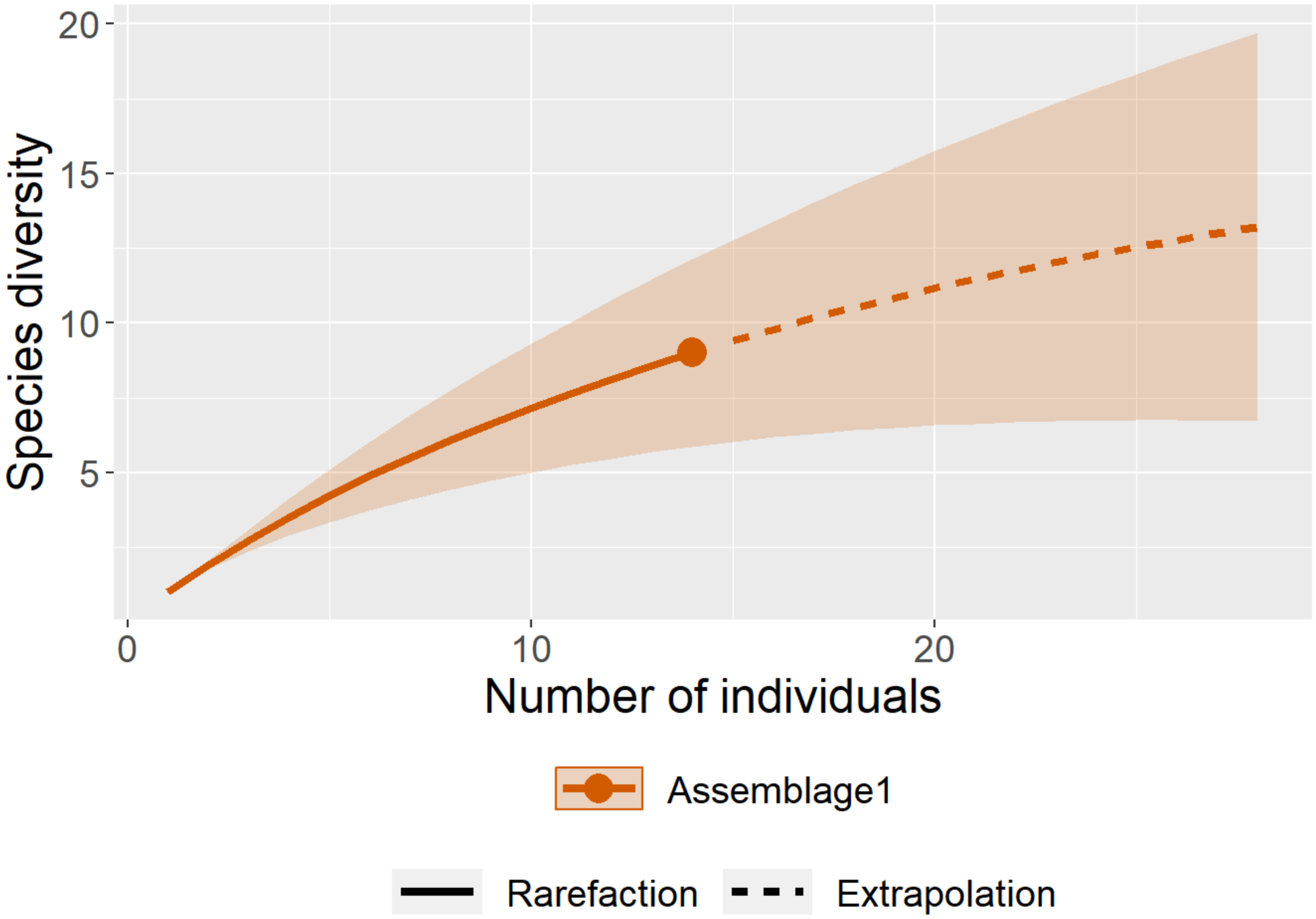


```
write.csv(as.list(m$data), "mData.csv")
```

plot coverage

```
c <- ggiNEXT(out, type=2)
```

```
## Warning in ggiNEXT.iNEXT(out, type = 2): invalid color.var setting, the iNEXT
## object do not consist multiple assemblages, change setting as Order.q
```

```
c
```

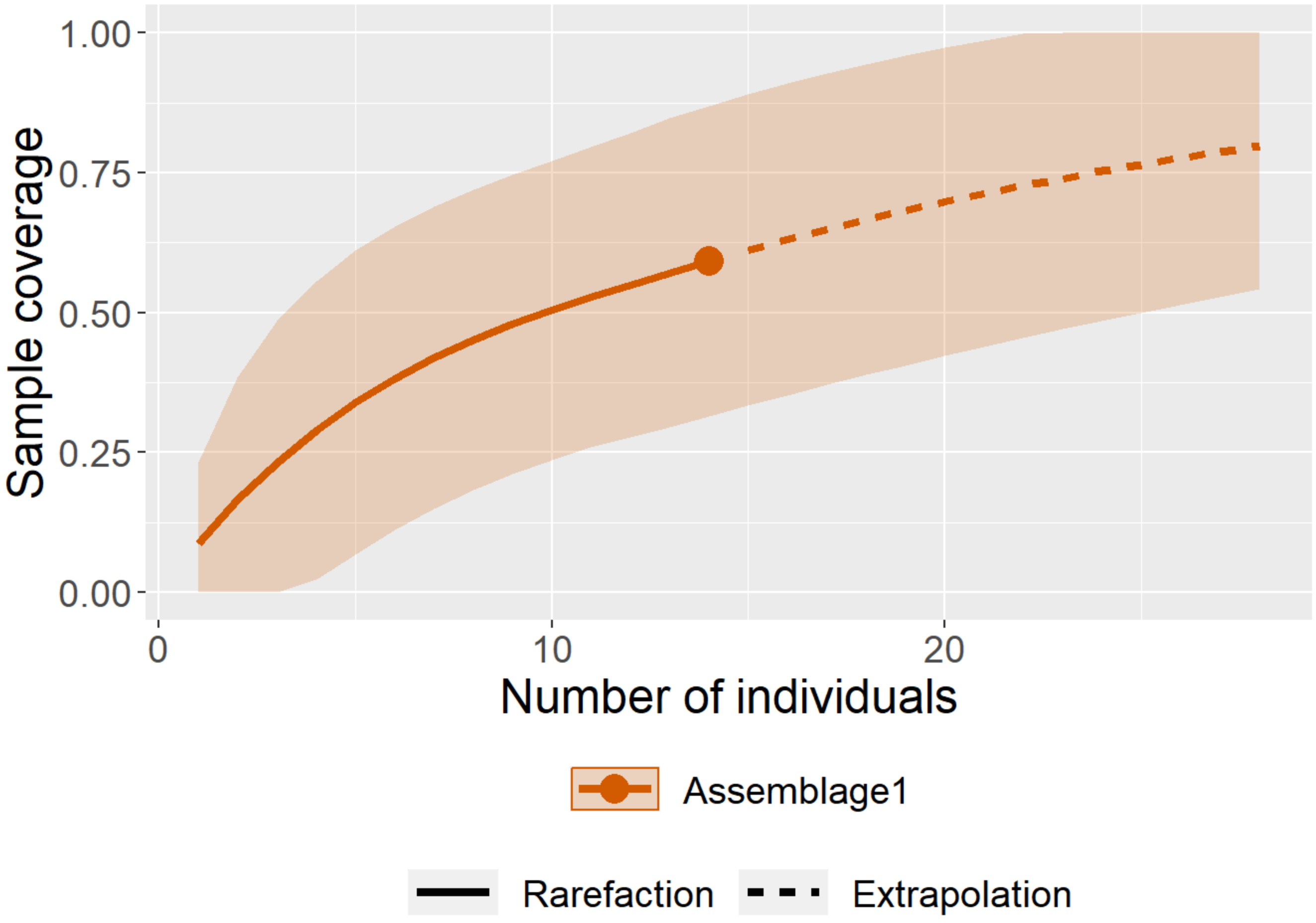


```
write.csv(as.list(c$data), "cData.csv")
```

evaulate FM with machine learning

```
FMsort16 <- (featureMatrix[ order(row.names(featureMatrix)), ])
mtuDF <- as.data.frame(SNRbruker$MTU)
colnames(mtuDF) <- c("species")
mtuFM <- cbind(mtuDF, FMsort16)
mtuFM$species <- as.factor(mtuFM$species)
# Rweka machne
m <- J48(species ~ ., data = mtuFM)
t <- table(predict(m), mtuFM$species)
Q <- summary(m)
head(Q$details)
```

```
##          pctCorrect         pctIncorrect      pctUnclassified
##         73.33333333          26.66666667           0.00000000
##               kappa    meanAbsoluteError rootMeanSquaredError
##          0.69072165           0.05333333           0.16329932
```

```
qD <- as.data.frame(t(Q$details))
FM <- as.data.frame(FMsort16)
z <- (predict(m, newdata = FM, type = 'probability'))
z
```

```
##                                                                               1
## j1370ba1 c("D1", "D2", "D3", "A5", "A6", "A7", "A8", "A9", "H6", "E11", "E12")  0.3333333
## j1480ba3 c("B4", "B5", "B6", "B7", "H2", "E1", "E2", "E3")                      0.0000000
## j1570r2a1 c("B10", "B11", "B12", "B9", "H4", "E7", "E8")                        0.0000000
## j1663tsa1 c("D11", "D12", "D9", "C1", "C2", "C4", "C5", "C5", "C6")             0.0000000
## j1735sda2 c("A10", "A11", "A12", "C11", "C12", "D1", "D2", "H9", "F5", "F6")    0.3333333
## j1780r2a1 c("B5", "B7", "B8", "D4", "D5", "D6", "D7", "H7", "F1", "F2")         0.0000000
## j1781tsa1 c("B1", "B2", "B3", "D11", "D12", "D9", "E1", "H3", "E4", "E5", "E6") 0.0000000
## j1813sda1 c("H1", "H2", "H4", "E3", "E4", "E5", "E6", "C10", "C11", "C9")       0.0000000
## j1943r2a1 c("A5", "A7", "A8", "E11", "E7", "E8", "E9", "H5")                    0.3333333
## j2047tsa1 c("G1", "G3", "G5", "E12", "F1", "F2", "F3", "A10", "A11", "A9")      0.0000000
## j2069sda1 c("H10", "F7", "F8")                                                  0.0000000
## j2090tsa1 c("F1", "F2", "F4", "F5", "F6", "F8", "F9", "H8")                     0.0000000
## j2933tsa1 c("G10", "G11", "G9", "G4", "G5", "G6", "G7")                         0.0000000
## j2941sda1 c("C10", "C12", "C9", "G10", "G12", "G9")                             0.0000000
## j2987tsa1 c("E3", "E7", "E9")                                                   0.0000000
##                                                                               2
## j1370ba1 c("D1", "D2", "D3", "A5", "A6", "A7", "A8", "A9", "H6", "E11", "E12")  0
## j1480ba3 c("B4", "B5", "B6", "B7", "H2", "E1", "E2", "E3")                      0
## j1570r2a1 c("B10", "B11", "B12", "B9", "H4", "E7", "E8")                        1
## j1663tsa1 c("D11", "D12", "D9", "C1", "C2", "C4", "C5", "C5", "C6")             1
## j1735sda2 c("A10", "A11", "A12", "C11", "C12", "D1", "D2", "H9", "F5", "F6")    0
## j1780r2a1 c("B5", "B7", "B8", "D4", "D5", "D6", "D7", "H7", "F1", "F2")         0
## j1781tsa1 c("B1", "B2", "B3", "D11", "D12", "D9", "E1", "H3", "E4", "E5", "E6") 0
## j1813sda1 c("H1", "H2", "H4", "E3", "E4", "E5", "E6", "C10", "C11", "C9")       1
## j1943r2a1 c("A5", "A7", "A8", "E11", "E7", "E8", "E9", "H5")                    0
## j2047tsa1 c("G1", "G3", "G5", "E12", "F1", "F2", "F3", "A10", "A11", "A9")      0
## j2069sda1 c("H10", "F7", "F8")                                                  0
## j2090tsa1 c("F1", "F2", "F4", "F5", "F6", "F8", "F9", "H8")                     1
## j2933tsa1 c("G10", "G11", "G9", "G4", "G5", "G6", "G7")                         0
## j2941sda1 c("C10", "C12", "C9", "G10", "G12", "G9")                             0
## j2987tsa1 c("E3", "E7", "E9")                                                   0
##                                                                               3
## j1370ba1 c("D1", "D2", "D3", "A5", "A6", "A7", "A8", "A9", "H6", "E11", "E12")  0.3333333
## j1480ba3 c("B4", "B5", "B6", "B7", "H2", "E1", "E2", "E3")                      0.0000000
## j1570r2a1 c("B10", "B11", "B12", "B9", "H4", "E7", "E8")                        0.0000000
## j1663tsa1 c("D11", "D12", "D9", "C1", "C2", "C4", "C5", "C5", "C6")             0.0000000
## j1735sda2 c("A10", "A11", "A12", "C11", "C12", "D1", "D2", "H9", "F5", "F6")    0.3333333
## j1780r2a1 c("B5", "B7", "B8", "D4", "D5", "D6", "D7", "H7", "F1", "F2")         0.0000000
## j1781tsa1 c("B1", "B2", "B3", "D11", "D12", "D9", "E1", "H3", "E4", "E5", "E6") 0.0000000
## j1813sda1 c("H1", "H2", "H4", "E3", "E4", "E5", "E6", "C10", "C11", "C9")       0.0000000
## j1943r2a1 c("A5", "A7", "A8", "E11", "E7", "E8", "E9", "H5")                    0.3333333
## j2047tsa1 c("G1", "G3", "G5", "E12", "F1", "F2", "F3", "A10", "A11", "A9")      0.0000000
## j2069sda1 c("H10", "F7", "F8")                                                  0.0000000
## j2090tsa1 c("F1", "F2", "F4", "F5", "F6", "F8", "F9", "H8")                     0.0000000
## j2933tsa1 c("G10", "G11", "G9", "G4", "G5", "G6", "G7")                         0.0000000
## j2941sda1 c("C10", "C12", "C9", "G10", "G12", "G9")                             0.0000000
## j2987tsa1 c("E3", "E7", "E9")                                                   0.0000000
##                                                                               4
## j1370ba1 c("D1", "D2", "D3", "A5", "A6", "A7", "A8", "A9", "H6", "E11", "E12")  0
## j1480ba3 c("B4", "B5", "B6", "B7", "H2", "E1", "E2", "E3")                      1
## j1570r2a1 c("B10", "B11", "B12", "B9", "H4", "E7", "E8")                        0
## j1663tsa1 c("D11", "D12", "D9", "C1", "C2", "C4", "C5", "C5", "C6")             0
## j1735sda2 c("A10", "A11", "A12", "C11", "C12", "D1", "D2", "H9", "F5", "F6")    0
## j1780r2a1 c("B5", "B7", "B8", "D4", "D5", "D6", "D7", "H7", "F1", "F2")         1
## j1781tsa1 c("B1", "B2", "B3", "D11", "D12", "D9", "E1", "H3", "E4", "E5", "E6") 0
```

```
## j1813sda1 c("H1", "H2", "H4", "E3", "E4", "E5", "E6", "C10", "C11", "C9")      0
## j1943r2a1 c("A5", "A7", "A8", "E11", "E7", "E8", "E9", "H5")                    0
## j2047tsa1 c("G1", "G3", "G5", "E12", "F1", "F2", "F3", "A10", "A11", "A9")      0
## j2069sda1 c("H10", "F7", "F8")                                                  0
## j2090tsa1 c("F1", "F2", "F4", "F5", "F6", "F8", "F9", "H8")                     0
## j2933tsa1 c("G10", "G11", "G9", "G4", "G5", "G6", "G7")                         0
## j2941sda1 c("C10", "C12", "C9", "G10", "G12", "G9")                             0
## j2987tsa1 c("E3", "E7", "E9")                                                   0
##                                                                                 5
## j1370ba1 c("D1", "D2", "D3", "A5", "A6", "A7", "A8", "A9", "H6", "E11", "E12")  0
## j1480ba3 c("B4", "B5", "B6", "B7", "H2", "E1", "E2", "E3")                      0
## j1570r2a1 c("B10", "B11", "B12", "B9", "H4", "E7", "E8")                        0
## j1663tsa1 c("D11", "D12", "D9", "C1", "C2", "C4", "C5", "C5", "C6")             0
## j1735sda2 c("A10", "A11", "A12", "C11", "C12", "D1", "D2", "H9", "F5", "F6")    0
## j1780r2a1 c("B5", "B7", "B8", "D4", "D5", "D6", "D7", "H7", "F1", "F2")         0
## j1781tsa1 c("B1", "B2", "B3", "D11", "D12", "D9", "E1", "H3", "E4", "E5", "E6") 1
## j1813sda1 c("H1", "H2", "H4", "E3", "E4", "E5", "E6", "C10", "C11", "C9")      0
## j1943r2a1 c("A5", "A7", "A8", "E11", "E7", "E8", "E9", "H5")                    0
## j2047tsa1 c("G1", "G3", "G5", "E12", "F1", "F2", "F3", "A10", "A11", "A9")      0
## j2069sda1 c("H10", "F7", "F8")                                                  0
## j2090tsa1 c("F1", "F2", "F4", "F5", "F6", "F8", "F9", "H8")                     0
## j2933tsa1 c("G10", "G11", "G9", "G4", "G5", "G6", "G7")                         0
## j2941sda1 c("C10", "C12", "C9", "G10", "G12", "G9")                             1
## j2987tsa1 c("E3", "E7", "E9")                                                   0
##                                                                                         6
## j1370ba1 c("D1", "D2", "D3", "A5", "A6", "A7", "A8", "A9", "H6", "E11", "E12")  0.3333333
## j1480ba3 c("B4", "B5", "B6", "B7", "H2", "E1", "E2", "E3")                      0.0000000
## j1570r2a1 c("B10", "B11", "B12", "B9", "H4", "E7", "E8")                        0.0000000
## j1663tsa1 c("D11", "D12", "D9", "C1", "C2", "C4", "C5", "C5", "C6")             0.0000000
## j1735sda2 c("A10", "A11", "A12", "C11", "C12", "D1", "D2", "H9", "F5", "F6")    0.3333333
## j1780r2a1 c("B5", "B7", "B8", "D4", "D5", "D6", "D7", "H7", "F1", "F2")         0.0000000
## j1781tsa1 c("B1", "B2", "B3", "D11", "D12", "D9", "E1", "H3", "E4", "E5", "E6") 0.0000000
## j1813sda1 c("H1", "H2", "H4", "E3", "E4", "E5", "E6", "C10", "C11", "C9")      0.0000000
## j1943r2a1 c("A5", "A7", "A8", "E11", "E7", "E8", "E9", "H5")                    0.3333333
## j2047tsa1 c("G1", "G3", "G5", "E12", "F1", "F2", "F3", "A10", "A11", "A9")      0.0000000
## j2069sda1 c("H10", "F7", "F8")                                                  0.0000000
## j2090tsa1 c("F1", "F2", "F4", "F5", "F6", "F8", "F9", "H8")                     0.0000000
## j2933tsa1 c("G10", "G11", "G9", "G4", "G5", "G6", "G7")                         0.0000000
## j2941sda1 c("C10", "C12", "C9", "G10", "G12", "G9")                             0.0000000
## j2987tsa1 c("E3", "E7", "E9")                                                   0.0000000
##                                                                                   7
## j1370ba1 c("D1", "D2", "D3", "A5", "A6", "A7", "A8", "A9", "H6", "E11", "E12")  0.0
## j1480ba3 c("B4", "B5", "B6", "B7", "H2", "E1", "E2", "E3")                      0.0
## j1570r2a1 c("B10", "B11", "B12", "B9", "H4", "E7", "E8")                        0.0
## j1663tsa1 c("D11", "D12", "D9", "C1", "C2", "C4", "C5", "C5", "C6")             0.0
## j1735sda2 c("A10", "A11", "A12", "C11", "C12", "D1", "D2", "H9", "F5", "F6")    0.0
## j1780r2a1 c("B5", "B7", "B8", "D4", "D5", "D6", "D7", "H7", "F1", "F2")         0.0
## j1781tsa1 c("B1", "B2", "B3", "D11", "D12", "D9", "E1", "H3", "E4", "E5", "E6") 0.0
## j1813sda1 c("H1", "H2", "H4", "E3", "E4", "E5", "E6", "C10", "C11", "C9")      0.0
## j1943r2a1 c("A5", "A7", "A8", "E11", "E7", "E8", "E9", "H5")                    0.0
## j2047tsa1 c("G1", "G3", "G5", "E12", "F1", "F2", "F3", "A10", "A11", "A9")      0.5
## j2069sda1 c("H10", "F7", "F8")                                                  0.0
## j2090tsa1 c("F1", "F2", "F4", "F5", "F6", "F8", "F9", "H8")                     0.0
## j2933tsa1 c("G10", "G11", "G9", "G4", "G5", "G6", "G7")                         0.0
## j2941sda1 c("C10", "C12", "C9", "G10", "G12", "G9")                             0.0
## j2987tsa1 c("E3", "E7", "E9")                                                   0.5
```

```
##                                                                               8
## j1370ba1 c("D1", "D2", "D3", "A5", "A6", "A7", "A8", "A9", "H6", "E11", "E12")  0.0
## j1480ba3 c("B4", "B5", "B6", "B7", "H2", "E1", "E2", "E3")                      0.0
## j1570r2a1 c("B10", "B11", "B12", "B9", "H4", "E7", "E8")                        0.0
## j1663tsa1 c("D11", "D12", "D9", "C1", "C2", "C4", "C5", "C5", "C6")             0.0
## j1735sda2 c("A10", "A11", "A12", "C11", "C12", "D1", "D2", "H9", "F5", "F6")    0.0
## j1780r2a1 c("B5", "B7", "B8", "D4", "D5", "D6", "D7", "H7", "F1", "F2")         0.0
## j1781tsa1 c("B1", "B2", "B3", "D11", "D12", "D9", "E1", "H3", "E4", "E5", "E6") 0.0
## j1813sda1 c("H1", "H2", "H4", "E3", "E4", "E5", "E6", "C10", "C11", "C9")       0.0
## j1943r2a1 c("A5", "A7", "A8", "E11", "E7", "E8", "E9", "H5")                    0.0
## j2047tsa1 c("G1", "G3", "G5", "E12", "F1", "F2", "F3", "A10", "A11", "A9")      0.0
## j2069sda1 c("H10", "F7", "F8")                                                  0.5
## j2090tsa1 c("F1", "F2", "F4", "F5", "F6", "F8", "F9", "H8")                     0.0
## j2933tsa1 c("G10", "G11", "G9", "G4", "G5", "G6", "G7")                         0.5
## j2941sda1 c("C10", "C12", "C9", "G10", "G12", "G9")                             0.0
## j2987tsa1 c("E3", "E7", "E9")                                                   0.0
##                                                                               9
## j1370ba1 c("D1", "D2", "D3", "A5", "A6", "A7", "A8", "A9", "H6", "E11", "E12")  0.0
## j1480ba3 c("B4", "B5", "B6", "B7", "H2", "E1", "E2", "E3")                      0.0
## j1570r2a1 c("B10", "B11", "B12", "B9", "H4", "E7", "E8")                        0.0
## j1663tsa1 c("D11", "D12", "D9", "C1", "C2", "C4", "C5", "C5", "C6")             0.0
## j1735sda2 c("A10", "A11", "A12", "C11", "C12", "D1", "D2", "H9", "F5", "F6")    0.0
## j1780r2a1 c("B5", "B7", "B8", "D4", "D5", "D6", "D7", "H7", "F1", "F2")         0.0
## j1781tsa1 c("B1", "B2", "B3", "D11", "D12", "D9", "E1", "H3", "E4", "E5", "E6") 0.0
## j1813sda1 c("H1", "H2", "H4", "E3", "E4", "E5", "E6", "C10", "C11", "C9")       0.0
## j1943r2a1 c("A5", "A7", "A8", "E11", "E7", "E8", "E9", "H5")                    0.0
## j2047tsa1 c("G1", "G3", "G5", "E12", "F1", "F2", "F3", "A10", "A11", "A9")      0.5
## j2069sda1 c("H10", "F7", "F8")                                                  0.0
## j2090tsa1 c("F1", "F2", "F4", "F5", "F6", "F8", "F9", "H8")                     0.0
## j2933tsa1 c("G10", "G11", "G9", "G4", "G5", "G6", "G7")                         0.0
## j2941sda1 c("C10", "C12", "C9", "G10", "G12", "G9")                             0.0
## j2987tsa1 c("E3", "E7", "E9")                                                   0.5
##                                                                              10
## j1370ba1 c("D1", "D2", "D3", "A5", "A6", "A7", "A8", "A9", "H6", "E11", "E12")  0.0
## j1480ba3 c("B4", "B5", "B6", "B7", "H2", "E1", "E2", "E3")                      0.0
## j1570r2a1 c("B10", "B11", "B12", "B9", "H4", "E7", "E8")                        0.0
## j1663tsa1 c("D11", "D12", "D9", "C1", "C2", "C4", "C5", "C5", "C6")             0.0
## j1735sda2 c("A10", "A11", "A12", "C11", "C12", "D1", "D2", "H9", "F5", "F6")    0.0
## j1780r2a1 c("B5", "B7", "B8", "D4", "D5", "D6", "D7", "H7", "F1", "F2")         0.0
## j1781tsa1 c("B1", "B2", "B3", "D11", "D12", "D9", "E1", "H3", "E4", "E5", "E6") 0.0
## j1813sda1 c("H1", "H2", "H4", "E3", "E4", "E5", "E6", "C10", "C11", "C9")       0.0
## j1943r2a1 c("A5", "A7", "A8", "E11", "E7", "E8", "E9", "H5")                    0.0
## j2047tsa1 c("G1", "G3", "G5", "E12", "F1", "F2", "F3", "A10", "A11", "A9")      0.0
## j2069sda1 c("H10", "F7", "F8")                                                  0.5
## j2090tsa1 c("F1", "F2", "F4", "F5", "F6", "F8", "F9", "H8")                     0.0
## j2933tsa1 c("G10", "G11", "G9", "G4", "G5", "G6", "G7")                         0.5
## j2941sda1 c("C10", "C12", "C9", "G10", "G12", "G9")                             0.0
## j2987tsa1 c("E3", "E7", "E9")                                                   0.0
```

export summary of machine learning

```
write.csv(as.matrix(Q), "Qmjd.csv")
write.csv(as.matrix(z), "Zmjd.csv")
```

end of rmd
