# Supplementary material for: Resolution of MALDI-TOF compared to whole genome sequencing for identification of Bacillus species isolated from cleanrooms at NASA Johnson Space Center
Source: Front Microbiol. 2025 Apr 9;16:1499516. doi: 10.3389/fmicb.2025.1499516 (PMC12017291; doi:10.3389/fmicb.2025.1499516)
Supplement: Supplementary file 8 [file Data_Sheet_8.pdf]

Tukey Test Mass Spectra Similarities

Michael G. LaMontagne

2025-02-20

R Markdown

```
library(tidyverse)
```

```
## ── Attaching core tidyverse packages ──────────────────────── tidyverse 2.0.0 ──
## ✓ dplyr     1.1.4     ✓ readr     2.1.5
## ✓ forcats   1.0.0     ✓ stringr   1.5.1
## ✓ ggplot2   3.5.1     ✓ tibble    3.2.1
## ✓ lubridate 1.9.3     ✓ tidyr     1.3.1
## ✓ purrr     1.0.2
## ── Conflicts ────────────────────────────────────────── tidyverse_conflicts() ──
## ✕ dplyr::filter() masks stats::filter()
## ✕ dplyr::lag()    masks stats::lag()
## ℹ Use the conflicted package (<http://conflicted.r-lib.org/>) to force all conflicts to become errors
```

```
library(readxl)
library(ggplot2)
library(stringr)
library(dplyr)
library(ggplot2)
library(forcats)
library(agricolae)
sessionInfo()
```

```
## R version 4.4.1 (2024-06-14 ucrt)
## Platform: x86_64-w64-mingw32/x64
## Running under: Windows 11 x64 (build 22631)
##
## Matrix products: default
##
##
## locale:
## [1] LC_COLLATE=English_United States.utf8
## [2] LC_CTYPE=English_United States.utf8
## [3] LC_MONETARY=English_United States.utf8
## [4] LC_NUMERIC=C
## [5] LC_TIME=English_United States.utf8
##
## time zone: America/Chicago
## tzcode source: internal
##
## attached base packages:
## [1] stats     graphics  grDevices utils     datasets  methods   base
##
## other attached packages:
##  [1] agricolae_1.3-7 readxl_1.4.3    lubridate_1.9.3 forcats_1.0.0
##  [5] stringr_1.5.1   dplyr_1.1.4     purrr_1.0.2     readr_2.1.5
##  [9] tidyr_1.3.1     tibble_3.2.1    ggplot2_3.5.1   tidyverse_2.0.0
##
## loaded via a namespace (and not attached):
##  [1] sass_0.4.9        utf8_1.2.4        generics_0.1.3    stringi_1.8.4
##  [5] lattice_0.22-6    hms_1.1.3         digest_0.6.36     magrittr_2.0.3
##  [9] evaluate_1.0.1    grid_4.4.1        timechange_0.3.0  fastmap_1.2.0
## [13] cellranger_1.1.0  jsonlite_1.8.8    fansi_1.0.6       scales_1.3.0
## [17] jquerylib_0.1.4   cli_3.6.3         rlang_1.1.4       munsell_0.5.1
## [21] withr_3.0.2       cachem_1.1.0      yaml_2.3.10       tools_4.4.1
## [25] AlgDesign_1.2.1.1 tzdb_0.4.0        colorspace_2.1-0  vctrs_0.6.5
## [29] R6_2.5.1          lifecycle_1.0.4   MASS_7.3-60.2     cluster_2.1.6
## [33] pkgconfig_2.0.3   pillar_1.9.0      bslib_0.8.0       gtable_0.3.6
## [37] glue_1.7.0        xfun_0.45         tidyselect_1.2.1  rstudioapi_0.17.1
## [41] knitr_1.48        htmltools_0.5.8.1 nlme_3.1-164      rmarkdown_2.29
## [45] compiler_4.4.1
```

Import

```
AAIcos <- read_excel("AAIcosW.xlsx")
AAIcos <- as.data.frame(AAIcos)
saveRDS(AAIcos,"AAIcos.rds")
```

Set lineage

```
AAIcos$lineage <- as.factor(AAIcos$lineage)
```

Plot AAI

```
ggplot(AAIcos, aes(x = lineage, y = AAI)) +
  geom_violin(fill = NA) +
  geom_jitter(position = position_jitter(0.2)) +
  xlab("Lineage Shared") + ylab("Average Amino Acid Identity") + theme_bw() +
  theme(panel.grid.major = element_blank(), panel.grid.minor = element_blank()) +
  theme(legend.position = "right")
```

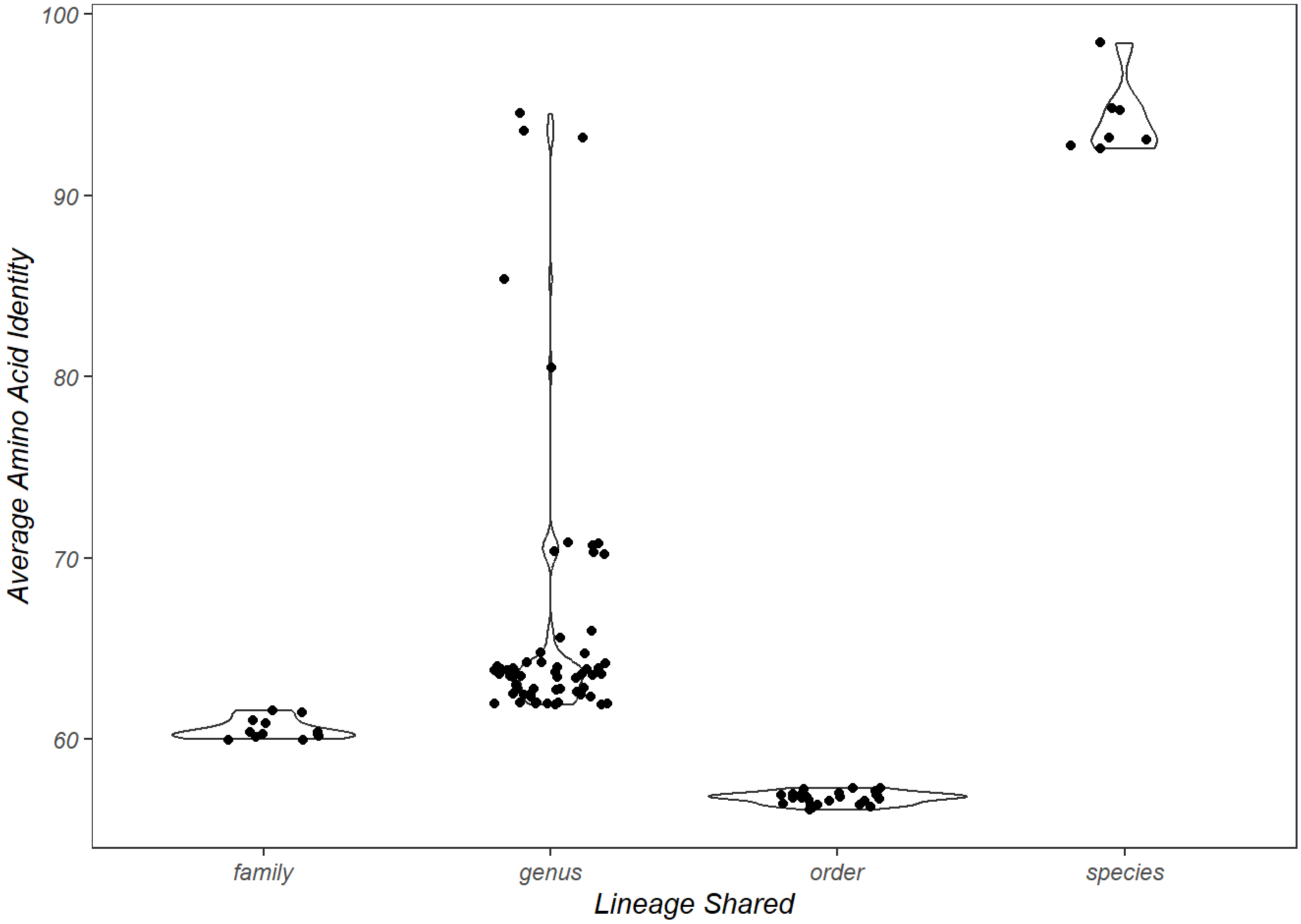

ANOVA test of AAI

```
fit <- aov(AAI ~ lineage, data=AAIcos)
summary(fit)
```

```
##              Df Sum Sq Mean Sq F value Pr(>F)
## lineage       3   8065  2688.4   78.32 <2e-16 ***
## Residuals   101   3467    34.3
## ---
## Signif. codes:  0 '***' 0.001 '**' 0.01 '*' 0.05 '.' 0.1 ' ' 1
```

Tukey test Jaccards

```
tukeyTest <- HSD.test(fit, trt = 'lineage', alpha = 0.0001)
tukeyTest
```

```
## $statistics
##    MSerror  Df     Mean      CV
##   34.32618 101 65.07048 9.00386
##
## $parameters
##    test  name.t ntr StudentizedRange alpha
##   Tukey lineage   4         6.385857 1e-04
##
## $means
##              AAI       std  r        se  Min  Max    Q25   Q50    Q75
## family  60.55833 0.5583390 12 1.6913057 60.0 61.6 60.175 60.35 60.925
## genus   66.17333 7.6309998 60 0.7563749 61.9 94.5 62.575 63.60 64.400
## order   56.75769 0.3372513 26 1.1490160 56.1 57.3 56.450 56.80 56.975
## species 94.22857 2.0401914  7 2.2144390 92.6 98.4 92.950 93.20 94.750
##
## $comparison
## NULL
##
## $groups
##              AAI groups
## species 94.22857      a
## genus   66.17333      b
## family  60.55833     bc
## order   56.75769      c
##
## attr(,"class")
## [1] "group"
```

Plot Jaccard Coefficients

```
ggplot(AAIcos, aes(x = lineage, y = distJ)) +
  geom_violin(fill = NA) +
  geom_jitter(position = position_jitter(0.2)) +
  xlab("Lineage Shared") + ylab("Jaccard Coefficients") + theme_bw() +
  theme(panel.grid.major = element_blank(), panel.grid.minor = element_blank()) +
  theme(legend.position = "right")
```

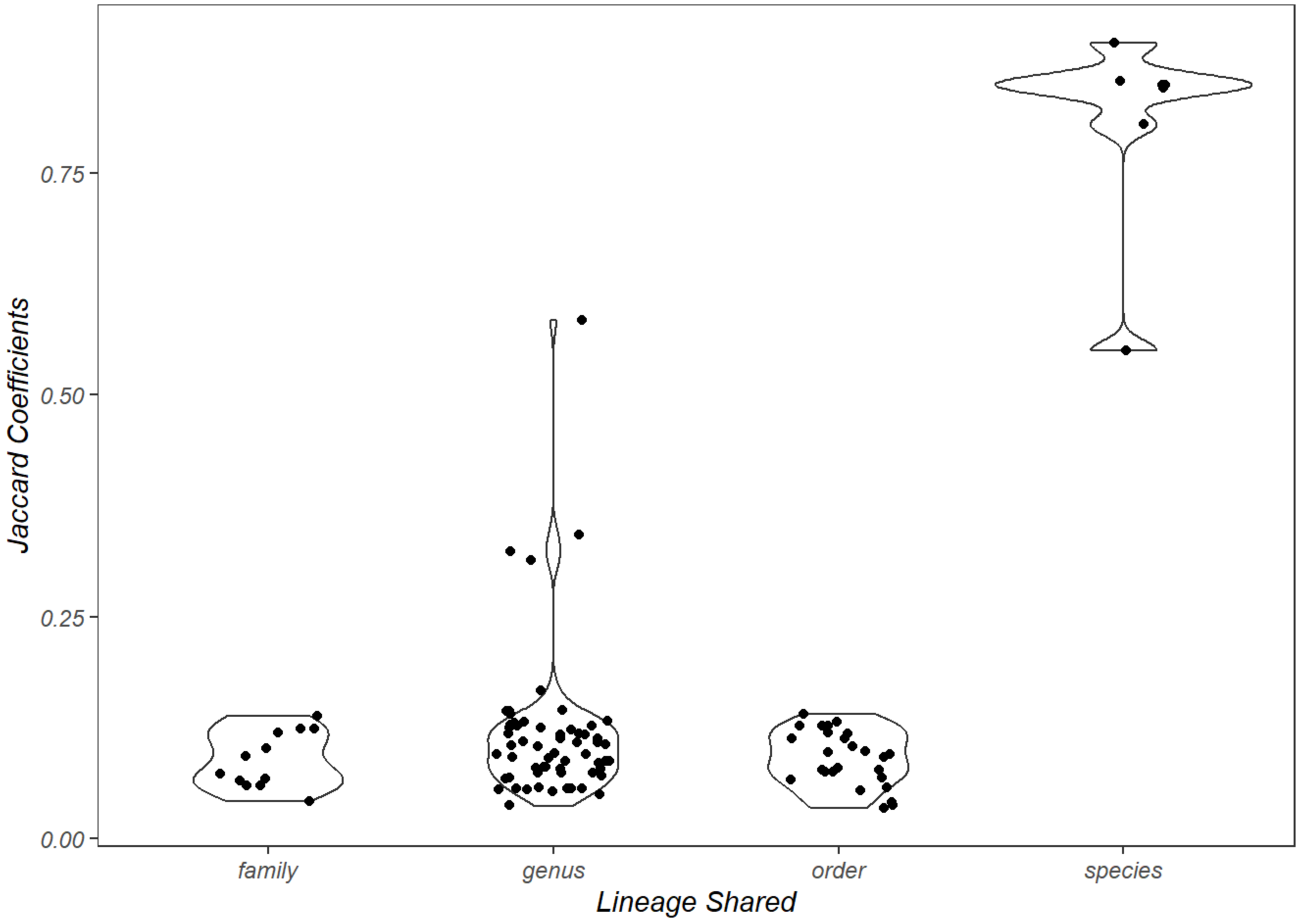

Plots cosine Similarities

```
ggplot(AAIcos, aes(x = lineage, y = distW)) +
  geom_violin(fill = NA) +
  geom_jitter(position = position_jitter(0.2)) +
  xlab("Lineage Shared") + ylab("Mass Spectra Similarity") + theme_bw() +
  theme(panel.grid.major = element_blank(), panel.grid.minor = element_blank()) +
  theme(legend.position = "right")
```

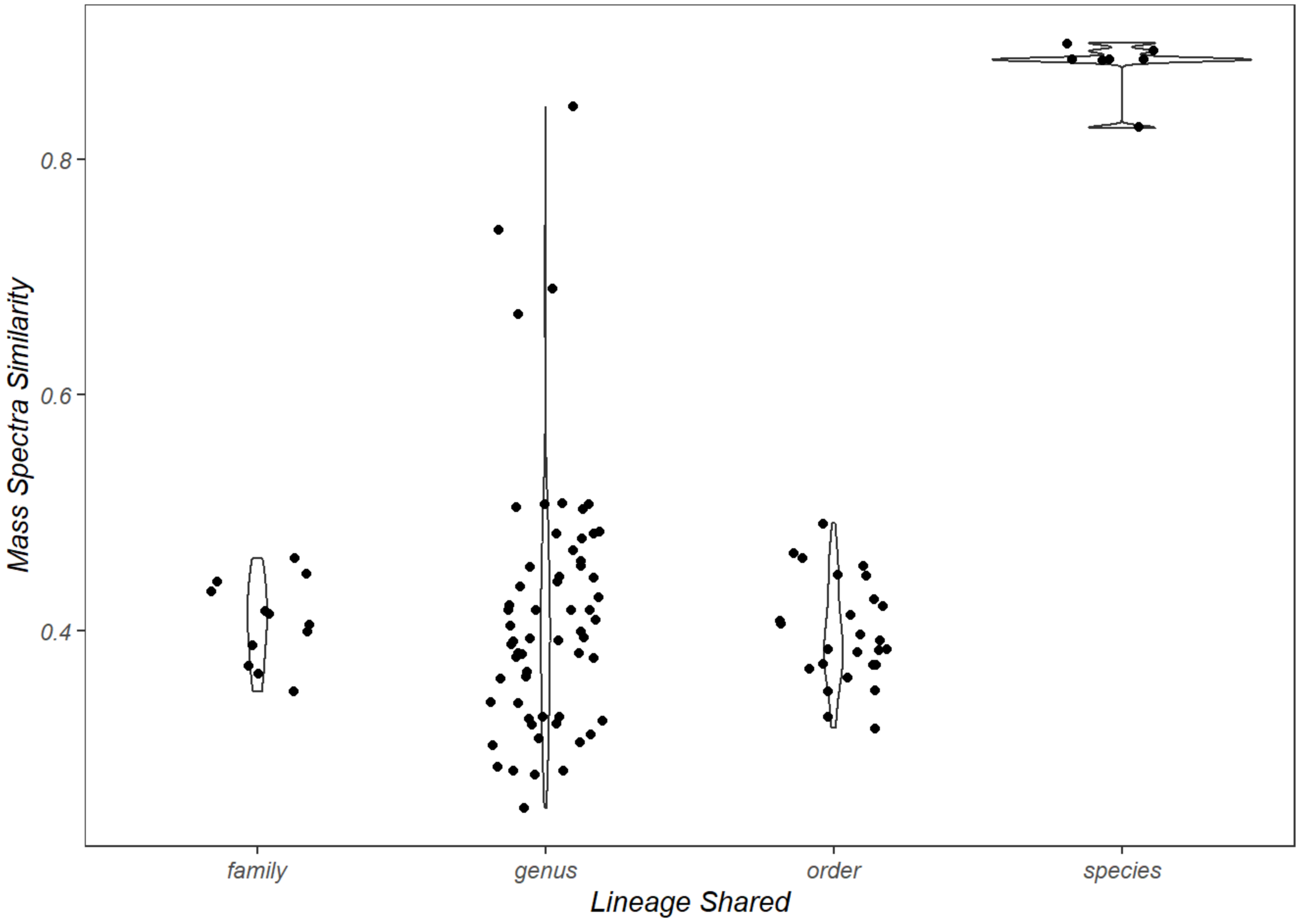

ANOVA test of Jaccard coefficients

```
fit <- aov(distJ ~ lineage, data=AAIcos)
summary(fit)
```

```
##              Df Sum Sq Mean Sq F value Pr(>F)
## lineage       3  3.232  1.0772   200.9 <2e-16 ***
## Residuals   101  0.542  0.0054
## ---
## Signif. codes:  0 '***' 0.001 '**' 0.01 '*' 0.05 '.' 0.1 ' ' 1
```

Tukey test Jaccards

```
tukeyTest <- HSD.test(fit, trt = 'lineage', alpha = 0.0001)
tukeyTest
```

```
## $statistics
##       MSerror  Df      Mean       CV
##   0.005361942 101 0.1526571 47.96715
##
## $parameters
##    test  name.t ntr StudentizedRange alpha
##   Tukey lineage   4         6.385857 1e-04
##
## $means
##              distJ        std  r          se   Min   Max    Q25   Q50     Q75
## family  0.08875000 0.03164613 12 0.021138319 0.042 0.138 0.0645 0.083 0.12000
## genus   0.11611667 0.08486741 60 0.009453344 0.037 0.585 0.0740 0.100 0.12550
## order   0.09019231 0.03099293 26 0.014360660 0.034 0.140 0.0705 0.093 0.11675
## species 0.80742857 0.11660454  7 0.027676556 0.550 0.897 0.8255 0.850 0.85200
##
## $comparison
## NULL
##
## $groups
##              distJ groups
## species 0.80742857      a
## genus   0.11611667      b
## order   0.09019231      b
## family  0.08875000      b
##
## attr(,"class")
## [1] "group"
```

ANOVA fit for cosine similarities

```
fit <- aov(distW ~ lineage, data=AAIcos)
summary(fit)
```

```
##              Df Sum Sq Mean Sq F value Pr(>F)
## lineage       3 1.4499  0.4833   61.53 <2e-16 ***
## Residuals   101 0.7933  0.0079
## ---
## Signif. codes:  0 '***' 0.001 '**' 0.01 '*' 0.05 '.' 0.1 ' ' 1
```

Tukey test cosinee similarities

```
tukeyTest <- HSD.test(fit, trt = 'lineage', alpha = 0.0001)
tukeyTest
```

```
## $statistics
##       MSerror  Df      Mean       CV
##   0.007854438 101 0.4406952 20.11033
##
## $parameters
##    test  name.t ntr StudentizedRange alpha
##   Tukey lineage   4         6.385857 1e-04
##
## $means
##             distW        std  r          se   Min   Max     Q25    Q50     Q75
## family  0.4070833 0.03543614 12 0.02558391 0.348 0.461 0.38275 0.4095 0.43500
## genus   0.4148000 0.11098829 60 0.01144147 0.249 0.845 0.33525 0.3965 0.45525
## order   0.3978077 0.04440542 26 0.01738085 0.317 0.491 0.37025 0.3880 0.42475
## species 0.8795714 0.02381776  7 0.03349720 0.827 0.899 0.88450 0.8850 0.88850
##
## $comparison
## NULL
##
## $groups
##             distW groups
## species 0.8795714      a
## genus   0.4148000      b
## family  0.4070833      b
## order   0.3978077      b
##
## attr(,"class")
## [1] "group"
```
