# Supplementary material for: Resolution of MALDI-TOF compared to whole genome sequencing for identification of Bacillus species isolated from cleanrooms at NASA Johnson Space Center
Source: Front Microbiol. 2025 Apr 9;16:1499516. doi: 10.3389/fmicb.2025.1499516 (PMC12017291; doi:10.3389/fmicb.2025.1499516)
Supplement: Supplementary file 11 [file Data_Sheet_11.docx]

| Supplemental file names and captions. | | |
| --- | --- | --- |
| File Name | Supplemental Material | Caption |
| S1A | Data Sheet 1.zip | Raw mass spectra A |
| S1B | Data Sheet 2.zip | Raw mass spectra B |
| S2 | Data Sheet 3.pdf | MALDIquant script in Rmarkdown format |
| S3 | Data Sheet 4.csv | Species names and spots  (speciesNamesQ.csv) |
| S4 | Data Sheet 5.zip | Alignment loop parameters (S014_DqJSCwgs2.Rds) |
| S5 | Data Sheet 6.csv | Species names and spots  (speciesNamesQ.csv) |
| S6 | Data Sheet 7.csv | Average amino acid identity (aaiJSC.csv) |
| S7 | Data Sheet 8.pdf | Tukey test of mass spectra similarities in Rmarkdown format |
| S8 | Image 1.tif | Jaccard coefficients for pairwise comparisons of mass spectra generated by MALDI-TOF |
| S9 | Image 3.tiff | Phylogenetic tree generated from 16S rRNA gene sequences |
| S10 | Image 2.tif | Rarefaction analysis |
| S11 | Data Sheet 9.zip | Raw spectra mass spectra in mzML format |
| S12 | Data Sheet 10.csv | Genes used to calculate average amino acid identity. |
| S12 | Table 1.docx | Strain names for reference sequences. |
