# Supplementary figures and images for: Resolution of MALDI-TOF compared to whole genome sequencing for identification of Bacillus species isolated from cleanrooms at NASA Johnson Space Center
Source: Front Microbiol. 2025 Apr 9;16:1499516. doi: 10.3389/fmicb.2025.1499516 (PMC12017291; doi:10.3389/fmicb.2025.1499516)

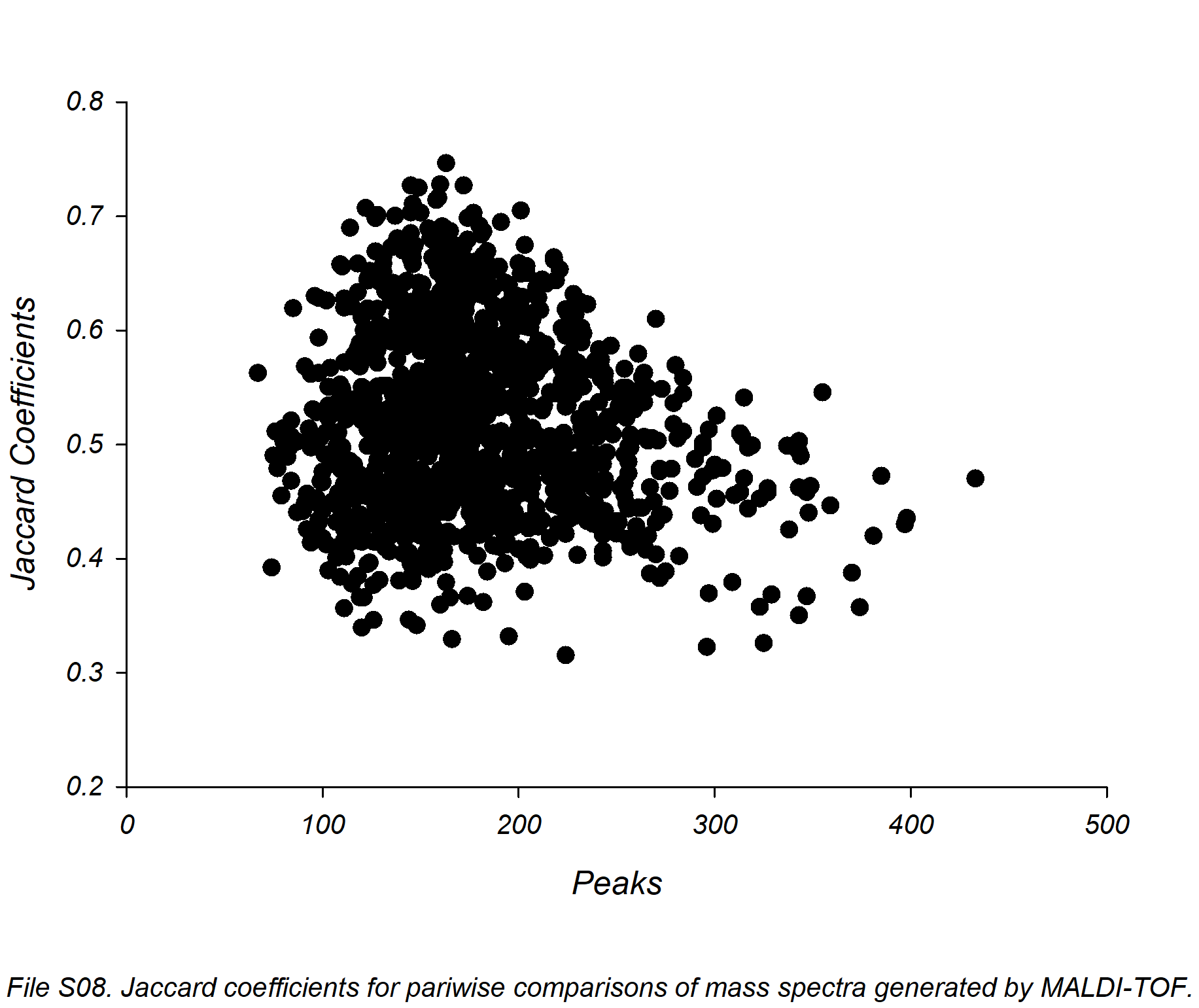

Supplement: Supplementary file 12 [file Image_1.tif]

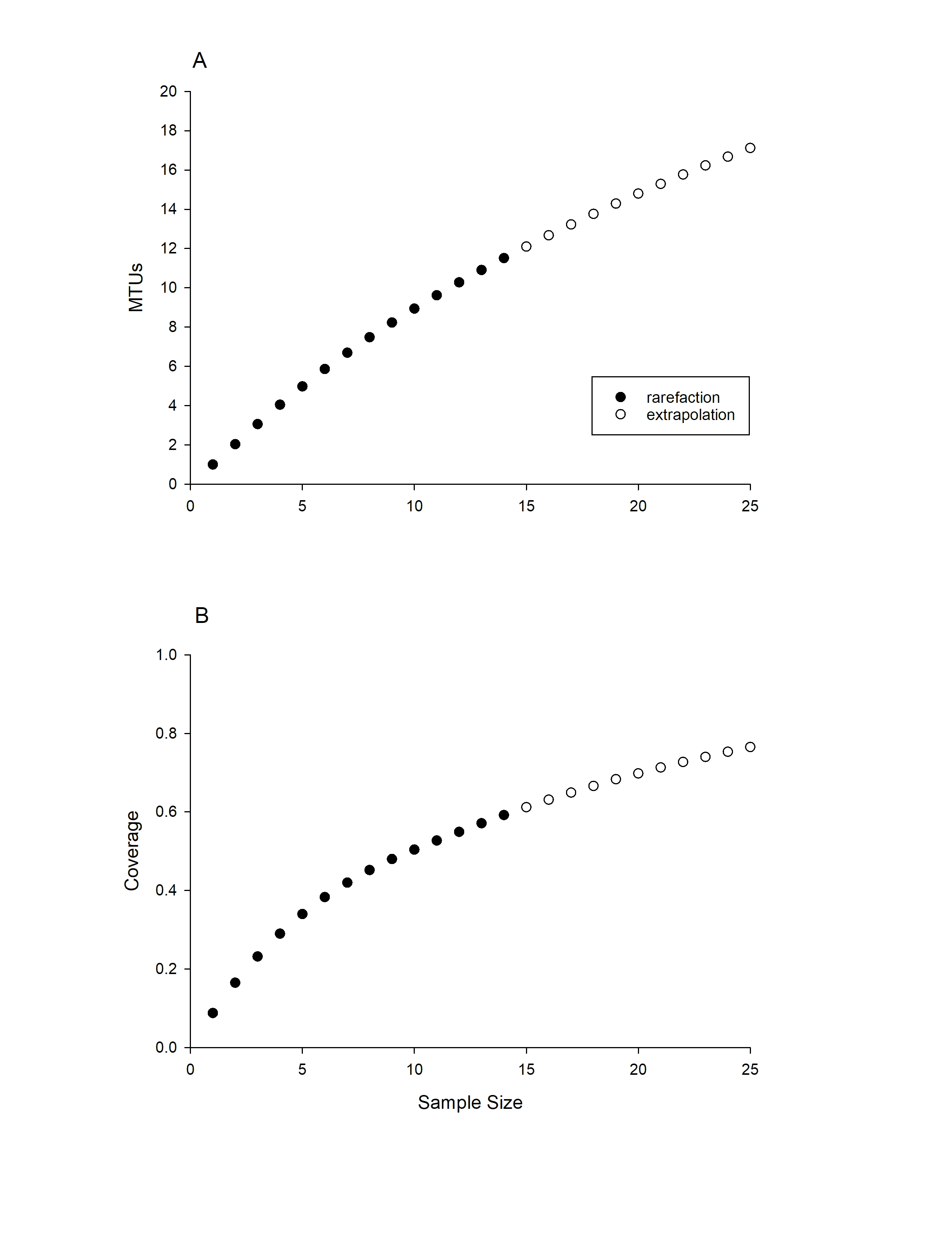

Supplement: Supplementary file 13 [file Image_2.tif]
