# Supplementary material for: Resolution of MALDI-TOF compared to whole genome sequencing for identification of Bacillus species isolated from cleanrooms at NASA Johnson Space Center
Source: Front Microbiol. 2025 Apr 9;16:1499516. doi: 10.3389/fmicb.2025.1499516 (PMC12017291; doi:10.3389/fmicb.2025.1499516)
Supplement: Supplementary file 15 [file Table_1.docx]

| **GenBank Assembly Number** | **Species Identification** | **Strain Name** |
| --- | --- | --- |
| GCA_000024285.1 | *Alicyclobacillus acidocaldarius* | DSM 446 |
| GCA_019749275.1 | *Paenibacillus xylanexedens* | PL-R3 |
| GCA_017874615.1 | *Paenibacillus xylanexedens* | DSM 21292 |
| GCA_001677985.1 | *Solibacillus silvestris* | MROC3 |
| GCA_023168345.1 | *Lysinibacillus species* | PLM2 |
| GCA_000473245.1 | *Bacillus infantis* | NRRL B-14911 |
| GCA_017303255.1 | *Priestia flexa* | GN22-4 |
| GCA_011405335.1 | *Bacillus wiedmannii* | PL1 |
| GCA_019749315.1 | *Bacillus cereus* | I1-R4 |
| GCA_013345915.1 | *Bacillus thuringiensis* | IIF4SW-P5 |
| GCA_009740005.1 | *Bacillus albus* | DLOU-Yingkou |
| GCA_003612955.1 | *Bacillus mobilis* | ML-A2C4 |
| GCA_018741285.1 | *Bacillus wiedmannii* | JAS07/5 |
| GCA_003866015.1 | *Bacillus wiedmannii* | FCC41 |
| GCA_019037245.1 | *Bacillus licheniformis* | 179-J 6A2 HS |
| GCA_019037645.1 | *Bacillus subtilis* | 179-F 1A1 HS |
| GCA_019748655.1 | *Bacillus velezensis* | S/N-302-OC-B3 |
| GCA_019749155.1 | *Bacillus velezensis* | J3-P2 |
| GCA_019749135.1 | *Bacillus velezensis* | JS3-R4 |
| GCA_019037745.1 | *Bacillus altitudinis* | 179-D 10A1 HS |
| GCA_019037045.1 | *Bacillus pumilus* | 179-K 7B4 HS |
| GCA_014267515.1 | *Bacillus pumilus* | 39M-5 |
| GCA_008244765.1 | *Bacillus safensis* | PgKB20 |
| GCA_019037495.1 | *Bacillus safensis* | 179-I 2B2 HS |
| GCA_019037445.1 | *Bacillus safensis* | 179-I 2B1 HS |
